# Performance and user acceptance of the Bhutan febrile and malaria information system: report from a pilot study

**DOI:** 10.1186/s12936-016-1105-0

**Published:** 2016-01-29

**Authors:** Tashi Tobgay, Pema Samdrup, Thinley Jamtsho, Kylie Mannion, Leonard Ortega, Amnat Khamsiriwatchara, Ric N. Price, Kamala Thriemer, Jaranit Kaewkungwal

**Affiliations:** Khesar Gyalpo University of Medical Sciences of Bhutan (KGUMSB), Changzamtog, P.O Box 446, Thimphu, Bhutan; Vector- Borne Disease Control Programme (VDCP), Ministry of Health, Thimphu, Bhutan; Public Health Laboratory, Department of Public Health, Ministry of Health, Thimphu, Bhutan; Global and Tropical Health Division, Menzies School of Health Research, Darwin, 0810 Australia; World Health Organization, SEARO, Indraprastha EstateMahatma Gandhi Marg, New Delhi, 110 002 India; Faculty of Tropical Medicine, Center of Excellence for Biomedical and Public Health Informatics (BIOPHICS), Mahidol University, 420/6 Ratchawithi Road, Bangkok, 10400 Thailand; Nuffield Department of Clinical Medicine, Centre for Tropical Medicine, University of Oxford, Oxford, UK

**Keywords:** Malaria, Bhutan, Surveillance, Mobile phone, GIS

## Abstract

**Background:**

Over the last decade, Bhutan has made substantial progress in controlling malaria. The country is now in an elimination phase, aiming to achieve no locally transmitted malaria by 2018. However, challenges remain and innovative control strategies are needed to overcome these. The evaluation and user acceptance of a robust surveillance tool applicable for informing malaria elimination activities is reported here.

**Methods:**

The Bhutan Febrile and Malaria Information System (BFMIS) is a combination of web-based and mobile technology that captures malariometric surveillance data and generates real time reports. The system was rolled out at six sites and data uploaded regularly for analysis. Data completeness, accuracy and data turnaround time were accessed by comparison to traditional paper based surveillance records. User acceptance and willingness for further roll out was assessed using qualitative and quantitative data.

**Results:**

Data completeness was nearly 10 % higher using the electronic system than the paper logs, and accuracy and validity of both approaches was comparable (up to 0.05 % in valid data and up to 3.06 % inaccurate data). Data turnaround time was faster using the BFMIS. General user satisfaction with the BFMIS was high, with high willingness of health facilities to adopt the system. Qualitative interviews revealed several areas for improvement before scale up.

**Conclusions:**

The BFMIS had numerous advantages over the paper-based system and based on the findings of the survey the Vector-Borne Disease Control Programme has taken the decision to incorporate the BMFIS and expand its use throughout all areas at risk for malaria as a key surveillance tool.

**Electronic supplementary material:**

The online version of this article (doi:10.1186/s12936-016-1105-0) contains supplementary material, which is available to authorized users.

## Background

Over the last decade, Bhutan has made substantial progress in controlling malaria. Between 2000 and 2013 the number of malaria cases fell from 5935 to 45 per year with malaria associated deaths decreasing from 15 to zero. Over the same period the annual parasite incidence (API) has fallen from 14 to 0.2/1000 risk population [1]. These gains prompted the country to move to an elimination phase, the target being no locally transmitted malaria by 2018 [2].

Despite a significant reduction of malaria incidence, over 50 % of the population remains at risk of malaria [3]. Movements across the southern border with India raise the threat of reintroduction of malaria into areas where it had been previously eliminated. Sustained vigilance is needed and in a remote and often inaccessible terrain, innovative control strategies are required [1]. Improvement in malaria case detection and surveillance is needed to identify outbreaks and target interventions appropriately.

The Vector-Borne Disease Control Programme (VDCP) in Bhutan currently collects weekly fever reports from all health centres. However, this paper-based data collection and collation is slow, often incomplete, contains less detailed data and does not provide timely reports that can inform control and elimination interventions. A recent review highlighted the need for strengthening case investigation and surveillance for all malaria species including mapping and reporting of all cases within 24 h in Bhutan [1]. An electronic reporting system based on mobile communication technology has significant advantages for ensuring real time reporting of complex data, if found to be reliable and well-accepted.

An electronic malaria information system (eMIS) has proven to be practical and reliable in Thailand [4], and may be particularly well suited to the remote malaria endemic regions of Bhutan. The eMIS was developed by the Center of Excellence for Biomedical and Public Health Informatics (BIOPHICS) [5] of the Faculty of Tropical Medicine, Mahidol University in Thailand in collaboration with Thai Ministry of Public Health. The Bhutan VDCP set out to develop a comparable system (the Bhutan Febrile-Malaria Information System, BFMIS) adapted to provide real time fever incidence rates as well as tools for spatial and temporal mapping of malaria cases.

In this paper, the results of a pilot study to assess the data quality and turnaround time of the BFMIS compared to the current paper based surveillance system as well as user acceptance and feasibility of scale up are reported.

## Methods

### Current surveillance system

Surveillance of febrile cases and malaria cases in Bhutan currently relies on a paper-based system. Data are collected at Basic Health Units (BHUs) and hospitals throughout the country, the number of febrile cases is summarized for each BHU on a weekly basis and transmitted as aggregated data to the VDCP using postal services or fax. Additional details for patients with malaria, are recorded in “malaria paper forms”, and also submitted to the VDCP for data entry into an electronic system at the central office. Each BHU generates monthly reports on febrile and malaria patients.

### Bhutan Febrile and Malaria Information System (BFMIS)

**Fig. 1:**
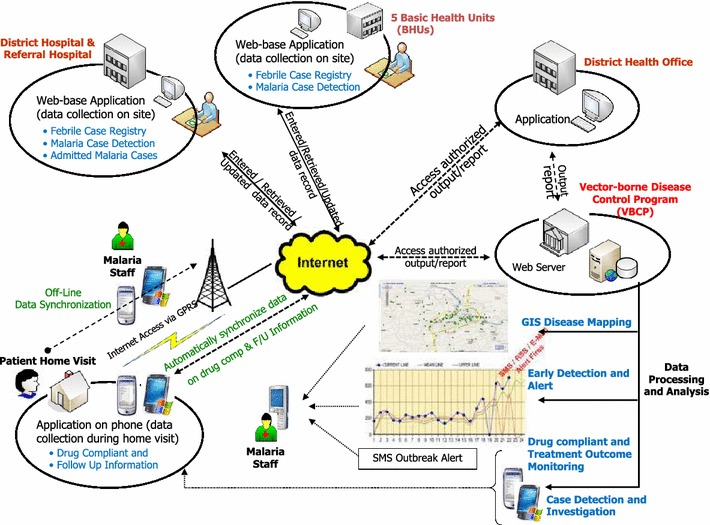
Overview of the BFMIS

The BFMIS consists of four main applications (Fig. 1):

1. The System Administration Application documents the administrative structure and organization charts.
2. The System User Applications (Table 1) assist the health care staff with case detection and patient management. It contains four modules:
  - The “Case registration module” collects data of new episodes of all febrile patients who seek treatment at BHUs. This module is comparable to the information on febrile patients collected in the paper-based system.
  - The “Case notification module” collects patient information of confirmed malaria cases. This module is comparable to the information on malaria patients collected in the paper-based system.
  - The “Case investigation module” links the case notification module to a follow-up application on mobile phones. This module exceeds the data collected in the paper-based system.
  - The “Case follow-up module” collects follow-up data from malaria cases. This module exceeds the data collected in the paper-based system.

**Table 1:** Components of system user application

Module	Variables collected
Case registration	Demographic data, diagnostic test result, treatment details for malaria cases
Case notification	Travel history, case classification
Case investigation	Source of infection, breeding sites, GPS co-ordinates of patient location and breeding sites, vector control intervention details
Case follow up	Clinical and parasitological follow up of day 3, 7, 14, 21 and 28, GPS coordinates of patient location during follow up
3. The Web-based application is designed for data collection and simple analysis. The information processed in the web-based system uses data collected through the “Case notification” and “Case investigation” modules and generates five standard output reports based on the WHO Indicators [4]:
  - annual summary of malaria cases
  - diagnosis of malaria cases by sites
  - fever cases and types of malaria infection by sites
  - monthly fever cases and types of malaria infection by sites
  - case management and follow-up by types of patient.
4. The Mobile phone application serves the collection of patient data during active follow up visits of field workers (follow up module). The module captures clinical details on the patient location and follow up visits, as well as coordinates of potential mosquito breeding sites associated with the episode. Different types of data (text, pictures, geo-referencing) can be captured through this application. Once patient follow up is completed, the data is uploaded via the mobile network to the central server and synchronized with the existing database. Examples of screen shots of the mobile application are shown in Additional file 1.

The BFMIS central server collates information from the field and generates reports in real time. These reports can be tailored to study sites including statistics on the type of patient, demographics and malaria case details. The system was developed using C Sharp language and Microsoft SQL Server 2005 with replication technology for backend databases. The XML and PHP language were used for developing web reports and mapping positive cases on google maps. The mobile application is compatible on Android mobile phones >4.0.0 OS.

### Implementation of the BFMIS

**Fig. 2:**
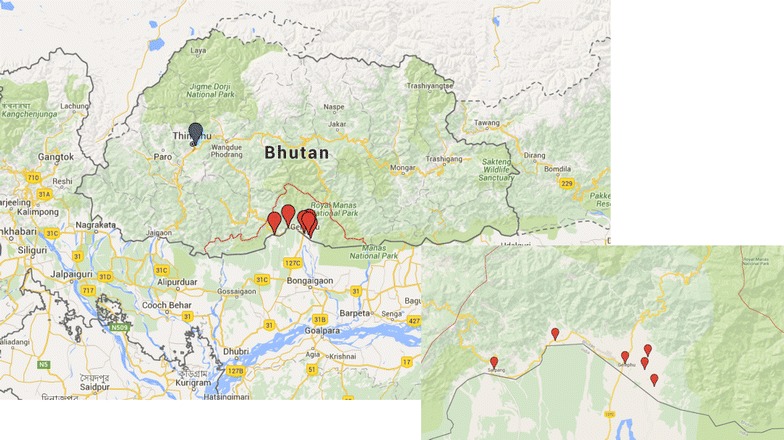
Map of the study sites

The pilot phase was implemented in the malaria endemic areas in Sarpang district an area of approximately 1655 km^2^ from January until December 2013. A total of six project sites (Fig. 2) were purposefully selected for initial role out due to their known high burden of disease and based on capacity of the site staff. Sarpang district alone covers more than 50 % of the countries malaria burden [1, 3]. All data entered through the web-based and the mobile applications were synchronized immediately after data collection to the national database centre, which is located in Thimphu (Fig. 2). During this phase the paper-based system was continued for comparison.

Local system users included health assistants and malaria technicians, who were trained over a 13-week period in March 2013. The development team at BIOPHICS, Bangkok provided a manual with clear instructions to end users at BHU or hospitals where the surveillance was conducted (Additional file 1).

### Assessment of data quality of the BFMIS

Assessment of data quality was performed in four of the six sites for variables collected in the case notification module and the case registration module in both systems in parallel. Data collected in the follow up module and case investigation module was not compared since only a fraction of those variables are collected in the paper-based system. Data from the BFMIS was cross-checked with the paper-based logs and the completeness, validity and accuracy of the data compared. Data completeness was defined as the lack of missing entries. Data validity was expressed as the percentage of illogical values defined as out of range values. Data accuracy referred to the consistency between the data in the paper-based logs and the data entered into the electronic system.

### Assessment of data turnaround time

Turnaround time was defined as the time elapsed from data collection for malaria cases until the data has reached the headquarters. Turnaround time between the BFMIS and the paper-based system was compared. Data upload within the BFMIS was done automatically when appropriate connection was available.

### Assessment of user acceptance and feasibility of scale up

Acceptability and feasibility for scale up of the BFMIS were assessed by both qualitative and quantitative analysis. The qualitative data were collected by an independent system monitoring team who visited four of the six study sites (two BHUs and two hospitals) and conducted semi-structured interviews with individual staff, who had been using the system for a minimum of 12 months. Interviews were conducted in local language and notes from the interviews were transcribed and compiled by topic area and coded. The interviews assessed the users experiences in using the system and any problems encountered. Satisfaction with the utility of the reporting was also documented as well as suggestions for system improvement. The interview team also visited potential sites for further scale up in a remote area to assess the feasibility of implementation and to interview staff regarding their readiness to use new technologies.

Quantitative data were collected through two different self-administered questionnaires. Form A (Additional file 2) was designed to collect data from the six current users to document acceptance and ease of use. Form B (Additional file 3) was designed to collect data from eight potential future users asking about the current working environment, computer systems, training experiences as well as the participants’ readiness to use new technologies at their working places. These data were used for a feasibility assessment to help decision-making on potential scale up to other sites beyond the current study sites. The respondents were asked to evaluate the desirability of features of a planned system rather than their attitudes towards the system and actual system use.

### Ethical approval

This research was approved by the Research Ethics Board for Health, Ministry of Health, Thimphu (approval letter no REBH/Approval/2012/010). An informed consent waiver was requested as the study was part of routine public health measures. Further there was no additional risk to the participants. The individual identifier information was not disclosed on the public domain excepted to the health officials required for carrying out the prevention and control activities. Considering this informed a consent waiver was granted by the Ethics Board.

## Results

### General

A total of 7141 individual patient records were entered into the case registration module of the BFMIS during the 1 year pilot phase at all of the six sites. These patients were all tested for suspected malaria and the 19 found to be positive by microscopy were entered into the case notification module (10 with *Plasmodium falciparum* and nine with *Plasmodium vivax*). All of these cases had entries in the case investigation module and the case follow up module.

### Data quality

#### Completeness

The number of cases registered in the case notification and the case registration modules were compared with the paper-based forms for each of four sites assessed (Table 2). Comparison of the number of malaria case records in the BFMIS case notification module and the paper log books showed consistency at all sites. The number of febrile cases recorded in the case registration module were between 3.8 and 9.8 % higher in the BFMIS compared to the paper log books. In the course of the implementation at the remaining site (Gelegphu) febrile cases where not reported systematically to the BFMIS due to the high workload of available staff (Table 2).

**Table 2:** Comparison of case counts from four sites in the BFMIS database and paper logbook, January–December 2013

Areas (GEOG sites)	Module	Cases in BFMIS database	Cases in logbook/paper report	Completeness of the BFMIS compared to the paper reports in %
Sarpang Hospital (Sompangkha)	Case registration module	4514	4350	+3.8 %
	Case notification module	6	6	Complete
Gelegphu regional Referral Hospital	Case registration module	94	4068	−97.7 %^a^
	Case notification module	8	8	Complete
Jigmaling (Chokorling)	Case registration module	1399	1367	+2.3 %
	Case notification module	1	1	Complete
Norbuling BHU (Sherzshong)	Case registration module	640	583	+9.8 %
	Case notification module	2	2	Complete

^a^Cases were not systematically entered into the BFMIS database due to high workload of staff caused by dual approach or registering patients in both systems

#### Validity and accuracy

A total of 580 records and 21 variables (12,180 data points) were used to assess validity and accuracy of the data in the case registration module. In total there were 60 (0.49 %) data values, which differed between the BFMIS and the paper logbook and a further four (0.03 %) data values were out of range or illogical in both sources. After assessing the records in the case notification module the inaccuracy rate rose to 3.06 % (18/589) with three (0.51 %) data points out of range or illogical.

### Data turnaround time

Although the target turnaround time with the paper based system was less than 15 days 37.8 % of the malaria records reached the headquarter after this time. In contrast, data collected using the BFMIS was transmitted immediately after data collection, with only minor delays (a matter of a few hours) for some cases due to issues around internet connectivity.

### User acceptance for the system

The quantitative questionnaires (Form A) were administered to six staff members at all of the sites responsible for the management of the BFMIS. Overall two third of users agreed or strongly agreed that the BFMIS was easy to use (Additional file 4), and 70 % of the users agreed or strongly endorsed the overall utility of the system (Additional file 5). Three users encountered minor reporting errors and two users had delays in processing time reported. The users identified several areas for improvements throughout the system including data menu functions and data entry and reporting (Additional file 6). Users at five of the six sites reported that the BFMIS was superior to the routine paper-based system, and also confirmed that the system was useful in generating appropriate information and reports (Additional file 7). Users at these sites were also keen to use the system, if it were continued.

Qualitative assessment showed that the system could have been improved if the system developers had consulted users before the development process. It should be noted that even though the development team went to collect system requirements prior to system design, not all study sites were visited.

### User feedback regarding scale up of the system

Eight sites were surveyed with regard to future roll out. Computers were available at all sites with internet access available at most of these. All personnel, both in the remote small BHU and the large hospital had more than 2 years of computer experience and used computers regularly. Though none were formally trained in information technology most had certain training(s) on computer usage.

All staff surveyed requested training on the management of electronic records, if the system were to be implemented at their sites (Table 3). One health worker highlighted that although the system could be useful, it should preclude continuation of essential training of malaria management and prevention.

**Table 3:** Experiences and needs assessment for computer use at potential sites (N = 8)

Experiences and needs	n (%)
Experiences using computer	
>2 years	8 (100 %)
Frequency of computer use	
Several times each day	7 (87.5 %)
Experiences in computer training	
Formal school computer and related course	0 (0.0 %)
Formal workshop, short course	1 (12.5 %)
Self-guided learning about computer	5 (62.5 %)
None	3 (37.5 %)
Basic ICT knowledge	
Correct knowledge score ≤25 %	3 (37.5 %)
Correct knowledge score 26–50 %	0 (0.0 %)
Correct knowledge score 51–75 %	3 (37.5 %)
Correct knowledge score ≥76 %	2 (25.0 %)
Use of mobile phone or tablets to collect data in healthcare work	
Ever used	5 (62.5 %)
Require training if e-Medical/Health record install	
Yes	8 (100 %)

## Discussion

The National Strategic Plan (2015–2020) has set its goal to achieve zero indigenous malaria in Bhutan by 2018 and obtain WHO-malaria-free certification by 2020 [2]. One of the components in the strategic plans is to strengthen surveillance systems, which in turn will provide evidence-based information for proper and in-time actions including diagnosis and treatment as well as vector control and other preventive measures. Mobile Health (mHealth) applications are of increasing interest for resource poor settings overcoming distances to remote areas and decreasing cost [6–11]. The BFMIS helped to strengthen the surveillance system by increasing data quality and turnaround time and facilitating active case detection around index cases through the geo-referencing tool.

There are several advantages inherent to the BFMIS compared to the paper based system. Repetitive data entry can be avoided and pre-specified data points can be generated automatically. Through mobile applications, data can be recorded from the actual locations during follow up and transmitted directly to the central server in real time. Data collected on mobile devices can capture textual data (as in paper-based) as well as locations (geo-referencing) and photos. Similar to other studies [6] data accuracy, validity and turnaround time in the BFMIS was high. Completeness of data was comparable between the electronic system and the paper-based logbooks, with the exception of the registration of febrile cases at one busy site, where this was not recorded systematically. Workload issues have been reported as one of the major obstacle in implementing any new system into the workplace in general [4], particularly, during the pilot phase of new systems when a dual approach is used [12].

Despite the high acceptance of the system and its potential for scale up, non-technical factors are the most significant barrier to its success [10, 13]. Many staff requested revision of outputs tailored to the needs of local health facilities and thus with greater operational applicability. This requires further system adjustment before expanding to other sites across the country. The recommendations for improvement of the current BFMIS system in regards to the menu and the data entry/management functions gathered in the interviews will be considered for the next version of the BFMIS.

One of the greatest risks for the success of the implementation of any electronic system is poor IT literacy of health workers [14]. This can be overcome by increased training in general and specifically for a new system [13]. IT staff from the central management level are needed to maintain database backups at different locations and to help conduct onsite individual trainings for health workers. Discussion with staff at some sites revealed that they could work more efficiently if there were locally based IT personnel rather than having to wait for IT help from central office causing delays. A more practical solution would be capacity building for healthcare personnel through development of IT and system management skills.

There are important limitations to this assessment. Firstly the new electronic system was piloted alongside the pre-existing paper system and data from the two systems was compared. This is a less robust than a formal comparison with intervention and control sites. Second, the study was not designed to compare costs between the paper based and the electronic system, however other studies [6, 7, 11] indicate that computerized data collection systems are more cost effective than paper based systems [10]. More studies are needed to assess cost-effectiveness formally.

The case registration module collects data on all febrile patients. With decreasing malaria numbers the aetiology and management of non-malaria febrile illness becomes increasingly important. The system therefore has the capacity to be extended to other febrile disease depending on available diagnostic tools and could be used to detect non-malaria febrile disease outbreaks.

## Conclusions

A web and mobile phone based surveillance system was implemented in six remote areas in Bhutan. Data accuracy, completeness, validity and turnaround time and user satisfaction were all high. Electricity and mobile network coverage is improving at a rapid pace in Bhutan. The BMFIS has the potential for expansion and will be part of the strategy of strengthening surveillance systems in moving towards the malaria elimination goal in 2018 under the Global Fund. Suggestions on improvements will be incorporated into a new version of the BFMIS before further scale-up.

## Additional files

10.1186/s12936-016-1105-0 Training manual.
10.1186/s12936-016-1105-0 Quantitative questionnaire assessing acceptance and ease of use of the BFMIS (Form A).
10.1186/s12936-016-1105-0 Quantitative questionnaire assessing future usage (Form B).
10.1186/s12936-016-1105-0 Perceived ease of use of the BFMIS.
10.1186/s12936-016-1105-0 Perceived usefulness of the BFMIS.
10.1186/s12936-016-1105-0 Feedback of BFMIS users at study sites.
10.1186/s12936-016-1105-0 Overall Attitude, Satisfaction and Acceptance Factors of System Users.
