# Supplementary material for: Performance and user acceptance of the Bhutan febrile and malaria information system: report from a pilot study
Source: Malar J. 2016 Jan 29;15:52. doi: 10.1186/s12936-016-1105-0 (PMC4731940; doi:10.1186/s12936-016-1105-0)
Supplement: Supplementary file 1 — 10.1186/s12936-016-1105-0 Training manual. [file 12936_2016_1105_MOESM1_ESM.pdf]

USER MANUAL
FOR
BHUTAN MALARIA SYSTEM

2013

Document name: User Manual_BHUTAN Malaria Application_V 1.1.doc
Created By: BIOPHICS (Center of Excellence for Biomedical and Public Health Informatics)

Contents

LOG-IN AND EXIT PROGRAM.. **3**
HOME... **4**

DATA ENTRY.. **4**
Case register.. 5
Case notification.. 7
Case investigation.. 9
Case follow-up.. 11

REPORT... **12**
Malaria case registration form.. 13
Malaria case notification form... 13
Malaria case investigate form.. 17
Malaria case follow-up form... 20
Fever report form.. 23

SYNCHRONIZATION... **24**

ABOUT.. **24**

Get to know menu bar

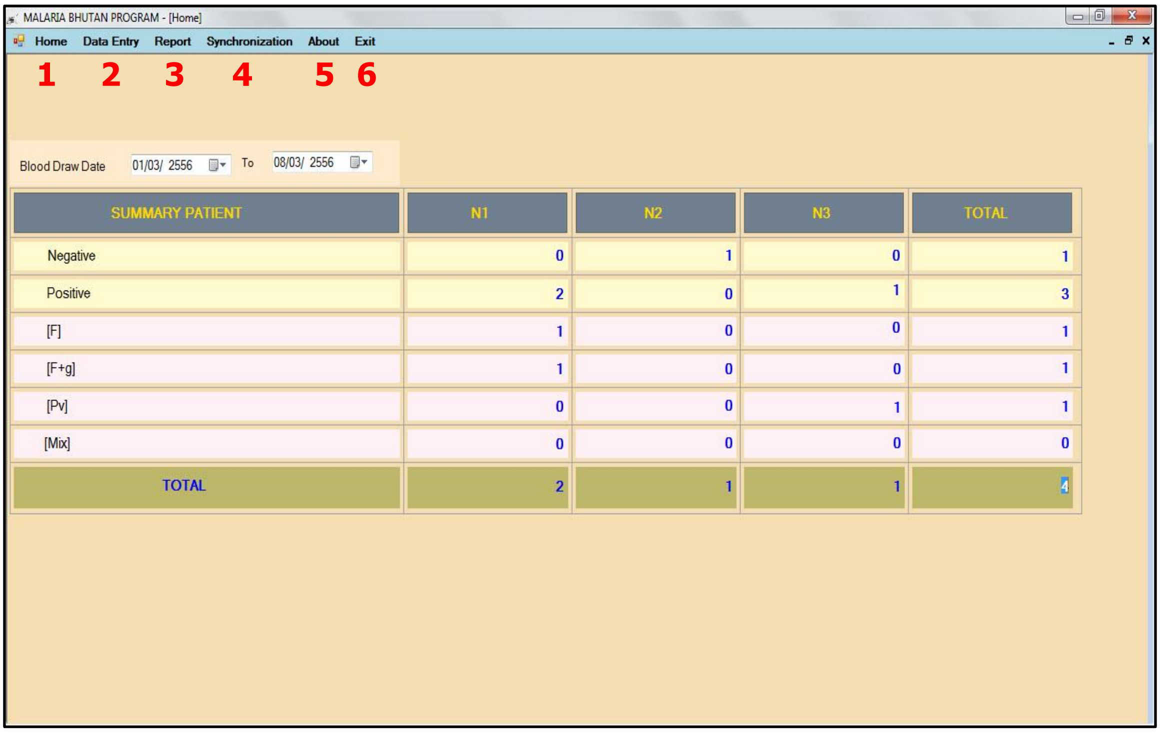

Number	Function	Sub-function
1	HOME	
2	Data Entry	- Case register<br>- Case notification<br>- Case investigation<br>- Case follow-up
3	Report	- Malaria case registration form<br>- Malaria case notification form<br>- Malaria case investigate form<br>- Malaria case follow-up form<br>- Fever report form
4	Synchronization	- Synchronize data
5	About	
6	Exit	

1. LOG-IN AND EXIT PROGRAM

1.1 Log-in program

Getting started Bhutan Malaria System, users need to log-in into system via username and password by following steps below:

1.1.1 Open the **"BHU Malaria System"** by double click icon below:

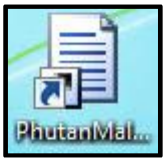

1.1.2 Fill username and password in the blank

1.1.3 Then, click log in key for getting started system (Figure 1)

***If username and password are correct, the **HOME** window will be opened (Figure 4).*

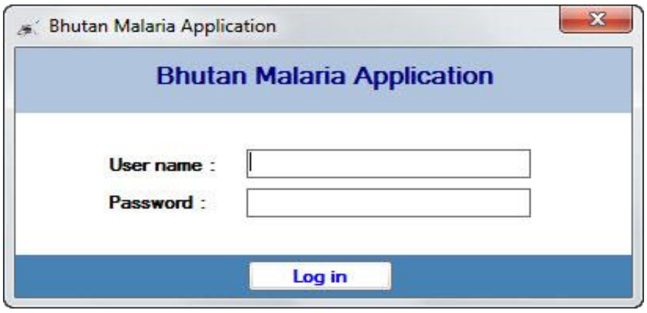

(Figure 1)

1.2 Exit program

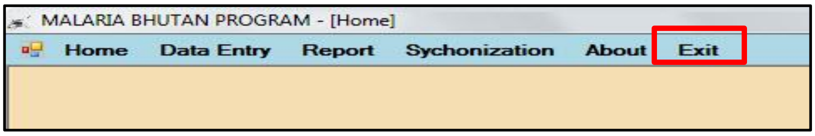

(Figure 2)

To exit the system, users can click the exit menu at menu bar (Figure 2) and then the system will show exit box for assuring to exit the system (Figure 3).

- Click **"Yes"** for exiting the system
- Click **"No"** to return into the system

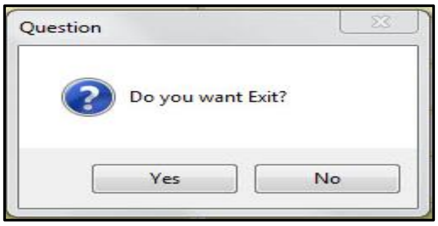

(Figure 3)

2. HOME

This window shows the summary of patient's number by malaria infection status and type of patients (Figure 4). Number of patients in **HOME** window will be showed depend on blood draw date. To see this window, user can click at **HOME** menu at menu bar then select blood draw date.

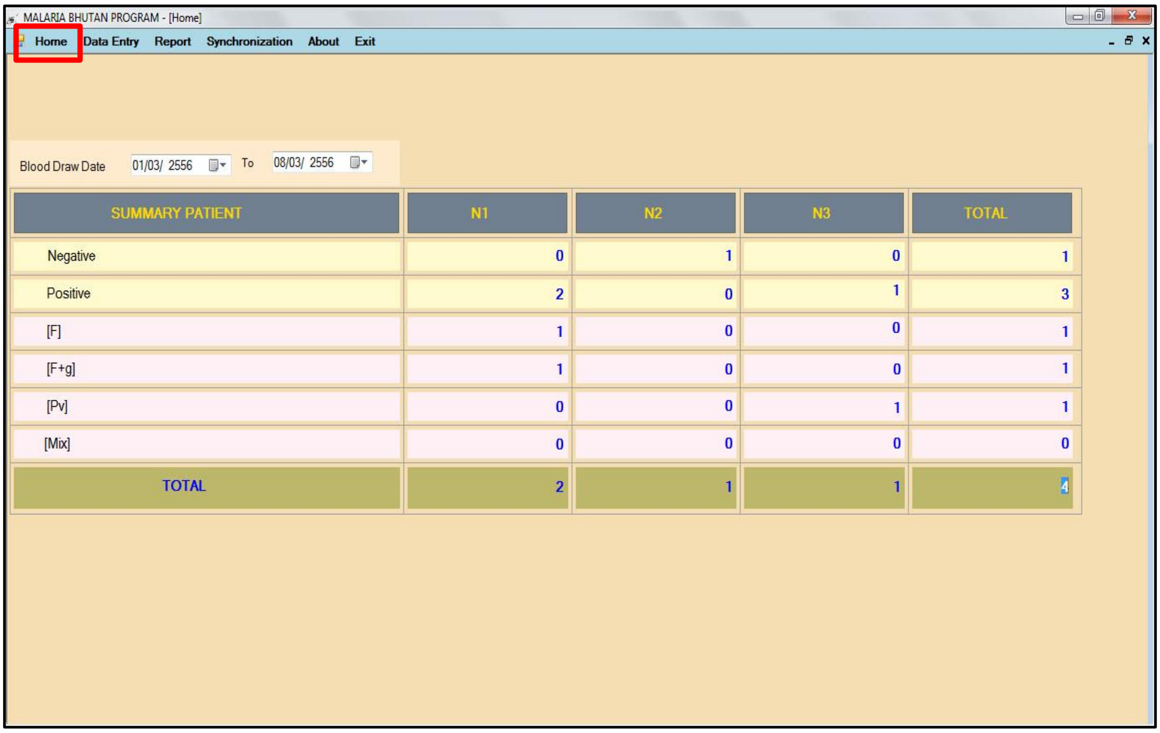

SUMMARY PATIENT	N1	N2	N3	TOTAL
Negative	0	1	0	1
Positive	2	0	1	3
[F]	1	0	0	1
[F+g]	1	0	0	1
[Pv]	0	0	1	1
[Mix]	0	0	0	0
TOTAL	2	1	1	4

(Figure 4)

3. DATA ENTRY

This menu will be used to register new patients and also enter the data for each form of malaria cases include malaria case register form, notification form, investigation form and follow up form. This menu composes of 4 sub-functions include;

- Case register
- Case notification
- Case investigation
- Case follow-up

3.1 Case register

Case register menu is a menu for adding data of new patients by following electronic form in the system. The index shows summary table of all patients who is already registered. Users can edit data or delete patients by using the edit and delete key at the end of patient's row in this summary table (Figure 5).

To register new patients, user can click at **"ADD DATA"** key (Figure 6) then the program will open the window of "malaria case register form" (Figure 7). This malaria case register form in the program is same as the paper form in Bhutan malaria routine work. Fill the patient's data in each blank and then click **"SAVE"** to register patient into the system.

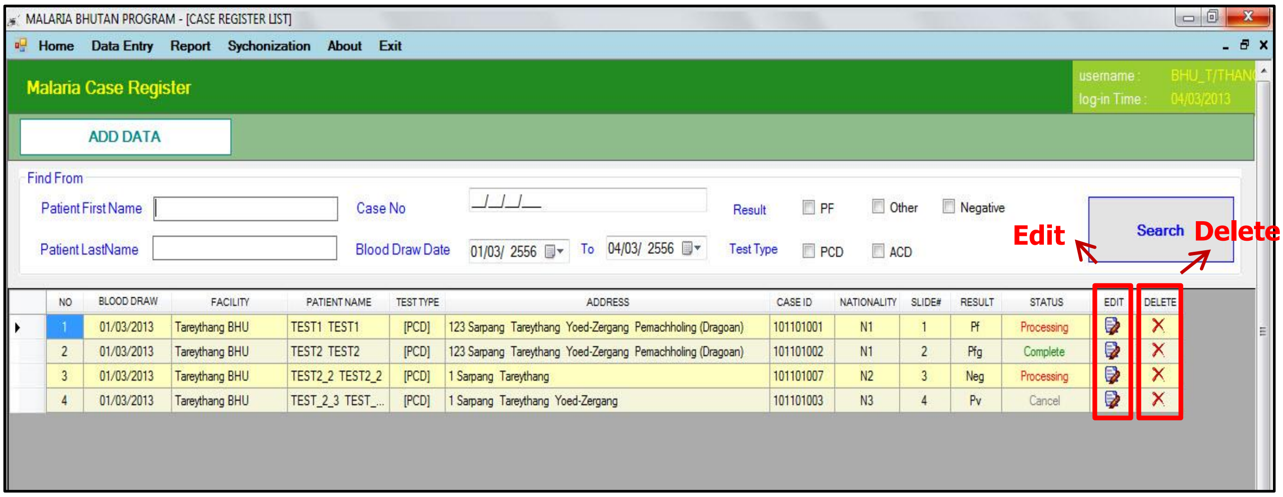

(Figure 5)

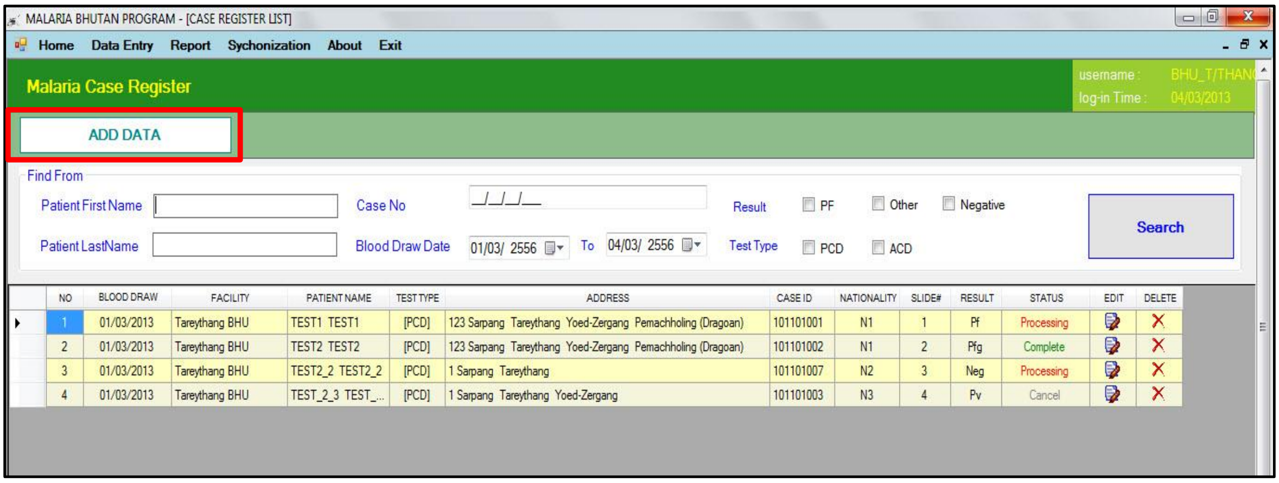

(Figure 6)

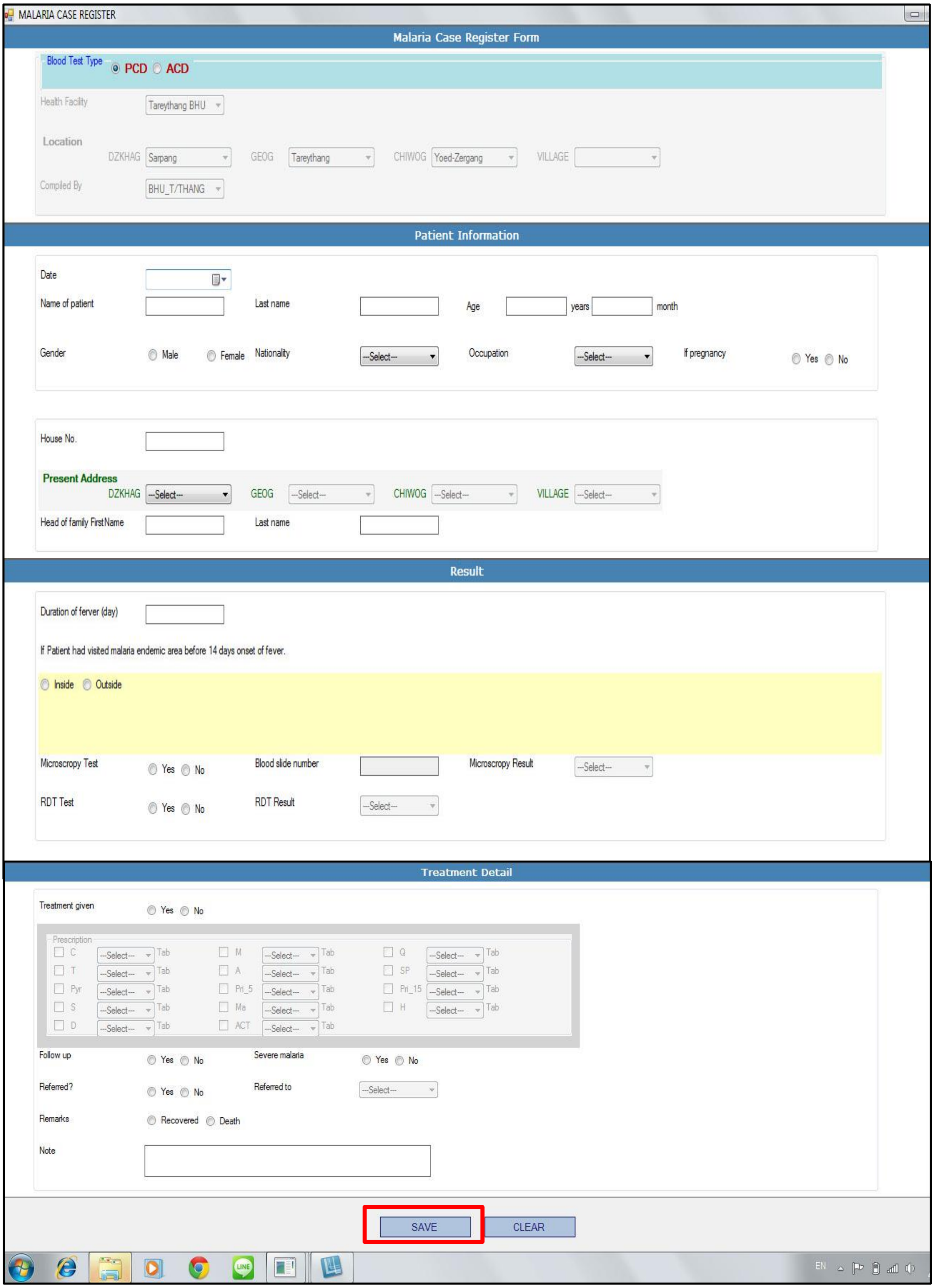
MALARIA CASE REGISTER
Malaria Case Register Form
Blood Test Type
PCD
ACD
Health Facility
Tareythang BHU
Location
DZKHAG
Sarpang
GEOG
Tareythang
CHIWOG
Yoed-Zergang
VILLAGE
Compiled By
BHU_T/THANG
Patient Information
Date
Name of patient
Last name
Age
years
month
Gender
Male
Female
Nationality
--Select--
Occupation
If pregnancy
Yes
No
House No.
Present Address
Head of family FirstName
Result
Duration of ferver (day)
If Patient had visited malaria endemic area before 14 days onset of fever.
Inside
Outside
Microscropy Test
Blood slide number
Microscropy Result
RDT Test
RDT Result
Treatment Detail
Treatment given
Prescription
C
T
Pyr
S
D
M
A
Pri_5
Ma
ACT
Q
SP
Pri_15
H
Tab
Follow up
Severe malaria
Referred?
Referred to
Remarks
Recovered
Death
Note
SAVE
CLEAR

(Figure 7)

3.2 Case notification

After users register patients in the system, the system will exclude patients who are positive results for malaria infection diagnosis into the list of notification. In the case notification menu, patients who are positive results will be listed in summary table (Figure 8). To fill data in the malaria case notification form, users can do it one by one by click **"ADD"** key at the end of patient's row in the table then the window of electronic malaria case notification form will be opened (Figure 10). Users fill the data in the fields include; patient information, history, and diagnosis and treatment, then click **"SAVE"** for completely form.

However, users can edit patients's form, cancle and delete patient's form by using function key at the end of patient's row in the table. To cancle patient's form, when users click **"CANCLE"** menu the system will ask you for reasons to cancle patient's form. The reasons to cancle patient's form include:

- *lost form*
- *don't get form*
- *already investigate but form not found*
- *other*

Users need to choose one reason before cancle patient's form and then click **"SAVE"** for completely cancle patient's form (Figure 9).

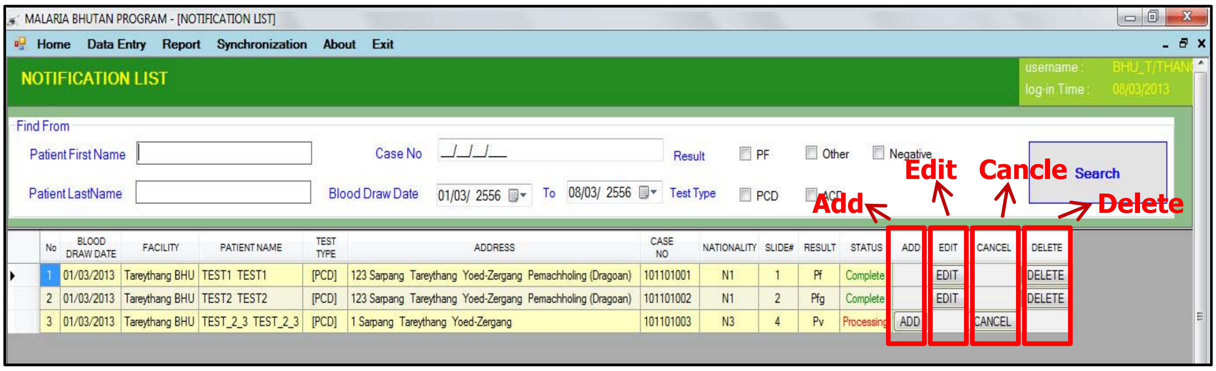

(Figure 8)

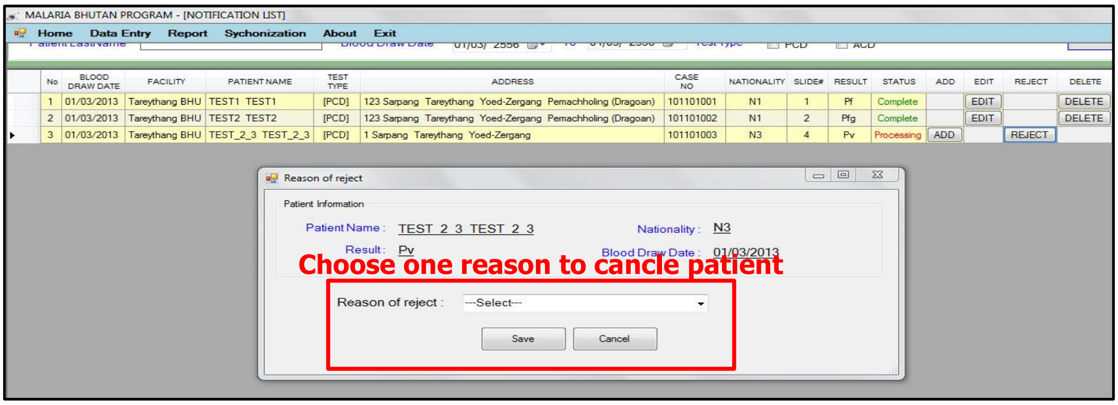

(Figure 9)

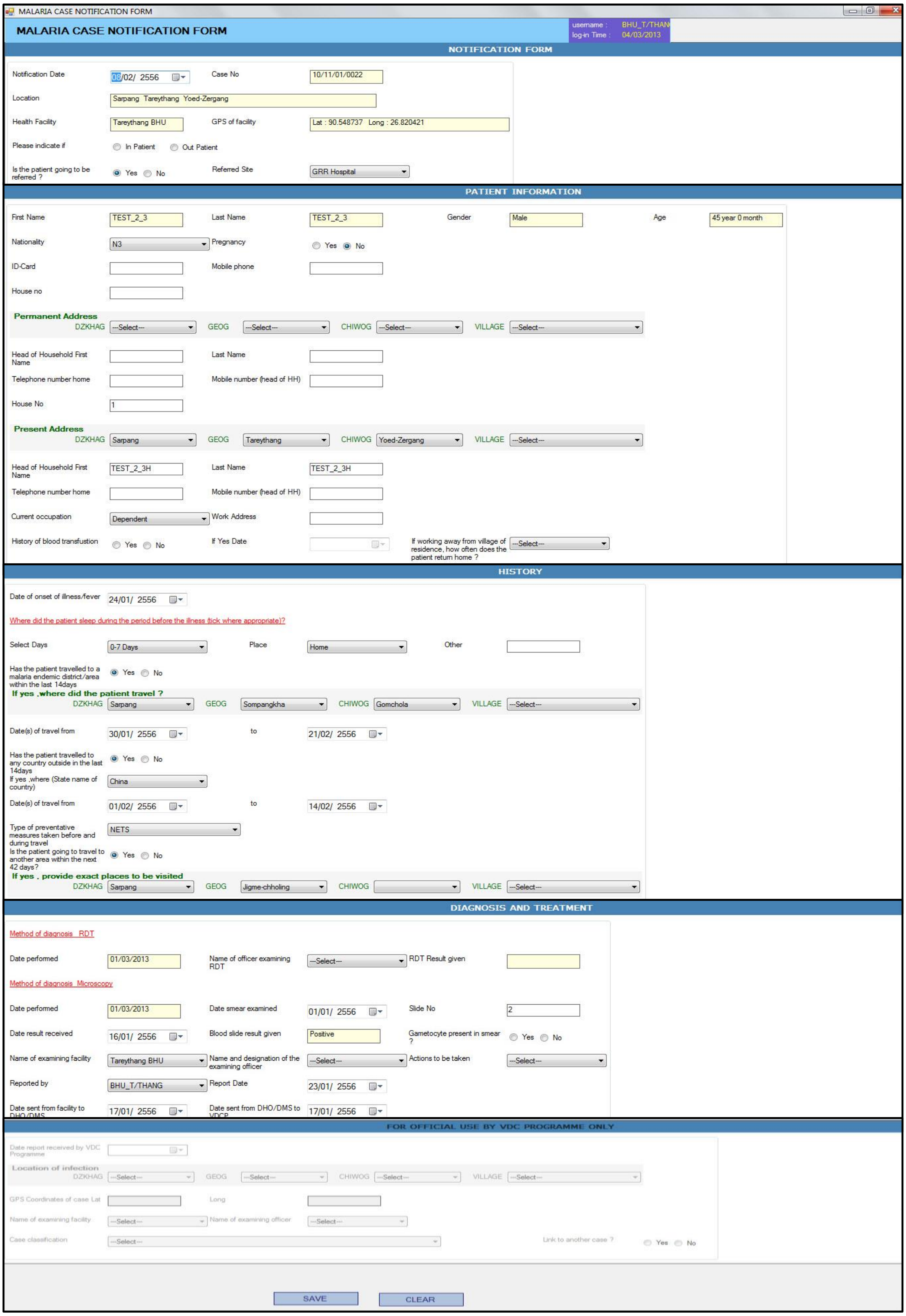
MALARIA CASE NOTIFICATION FORM
MALARIA CASE NOTIFICATION FORM
username : BHU_T/THAN
log-in Time : 04/03/2013
NOTIFICATION FORM
Notification Date 08/02/ 2556
Case No 10/11/01/0022
Location Sarpang Tareythang Yoed-Zergang
Health Facility Tareythang BHU
GPS of facility Lat : 90.548737 Long : 26.820421
Please indicate if In Patient Out Patient
Is the patient going to be referred ? Yes No
Referred Site GRR Hospital
PATIENT INFORMATION
First Name TEST_2_3
Last Name TEST_2_3
Gender Male
Age 45 year 0 month
Nationality N3
Pregnancy Yes No
ID-Card
Mobile phone
House no
Permanent Address
DZKHAG --Select--
GEOG --Select--
CHIWOG --Select--
VILLAGE --Select--
Head of Household First Name
Last Name
Telephone number home
Mobile number (head of HH)
House No 1
Present Address
DZKHAG Sarpang
GEOG Tareythang
CHIWOG Yoed-Zergang
VILLAGE --Select--
Head of Household First Name TEST_2_3H
Last Name TEST_2_3H
Telephone number home
Mobile number (head of HH)
Current occupation Dependent
Work Address
History of blood transfustion Yes No
If Yes Date
If working away from village of residence, how often does the patient return home ? --Select--
HISTORY
Date of onset of illness/fever 24/01/ 2556
Where did the patient sleep during the period before the illness (tick where appropriate)?
Select Days 0-7 Days
Place Home
Other
Has the patient travelled to a malaria endemic district/area within the last 14days Yes No
If yes ,where did the patient travel ?
DZKHAG Sarpang
GEOG Sompangkha
CHIWOG Gomchola
VILLAGE --Select--
Date(s) of travel from 30/01/ 2556 to 21/02/ 2556
Has the patient travelled to any country outside in the last 14days Yes No
If yes ,where (State name of country) China
Date(s) of travel from 01/02/ 2556 to 14/02/ 2556
Type of preventative measures taken before and during travel NETS
Is the patient going to travel to another area within the next 42 days? Yes No
If yes , provide exact places to be visited
DZKHAG Sarpang
GEOG Jigme-chholing
CHIWOG
VILLAGE --Select--
DIAGNOSIS AND TREATMENT
Method of diagnosis RDT
Date performed 01/03/2013
Name of officer examining RDT --Select--
RDT Result given
Method of diagnosis Microscopy
Date performed 01/03/2013
Date smear examined 01/01/ 2556
Slide No 2
Date result received 16/01/ 2556
Blood slide result given Positive
Gametocyte present in smear ? Yes No
Name of examining facility Tareythang BHU
Name and designation of the examining officer --Select--
Actions to be taken --Select--
Reported by BHU_T/THANG
Report Date 23/01/ 2556
Date sent from facility to DHO/DMS 17/01/ 2556
Date sent from DHO/DMS to VDCP 17/01/ 2556
FOR OFFICIAL USE BY VDC PROGRAMME ONLY
Date report received by VDC Programme
Location of infection
DZKHAG --Select--
GEOG --Select--
CHIWOG --Select--
VILLAGE --Select--
GPS Coordinates of case Lat
Long
Name of examining facility --Select--
Name of examining officer --Select--
Case classification --Select--
Link to another case ? Yes No
SAVE
CLEAR

(Figure 10)

3.3 Case investigation

This menu will be used to investigate patients by following the malaria case investigation form and this menu will be linked with follow-up application on mobile phone. List of patients in this menu are same as list of patients in the menu of case notification. Users can add the data for investigation one by one by click **"ADD"** key and also edit form, cancle and delete patient's form by using key at the end of patient's row (Figure 11). After users click **"ADD"** key to add investigation's data, the window of electronic malaria case investigation form will be opened (Figure 13). Users can fill data for patient's investigation by following fields in the form include; history of malaria control measures at patient's home, geographical reconnaissance information, entomological investigation and then click **"SAVE"** for completely form.

To cancle patient's form, when users click **"CANCLE"** menu the system will ask you for reasons to cancle patient's form. The reasons to cancle patient's form include:

- *lost form*
- *don't get form*
- *already investigate but form not found*
- *other*

Users need to choose one reason before cancle patient's form and then click **"SAVE"** for completely cancle patient's form (Figure 12).

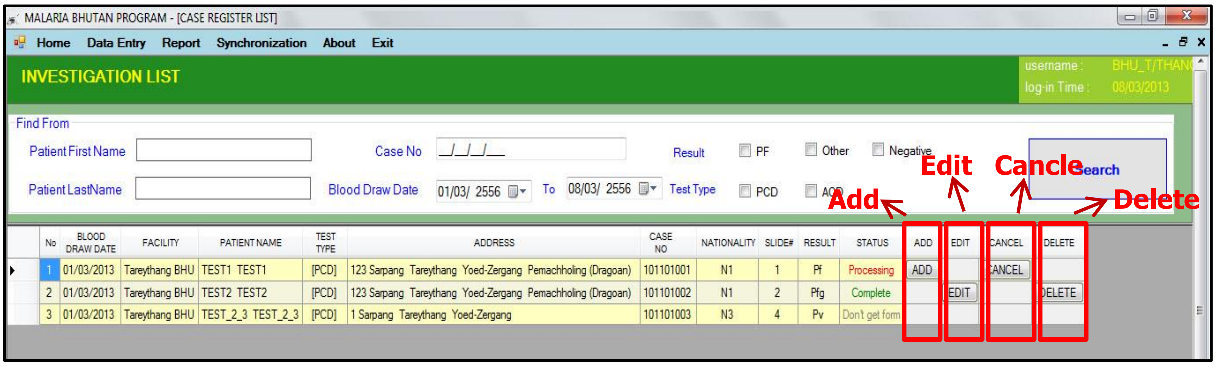

(Figure 11)

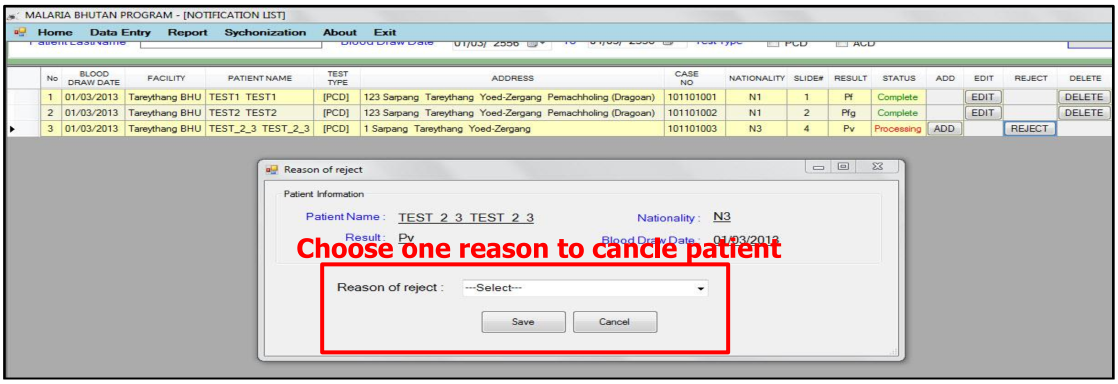

(Figure 12)

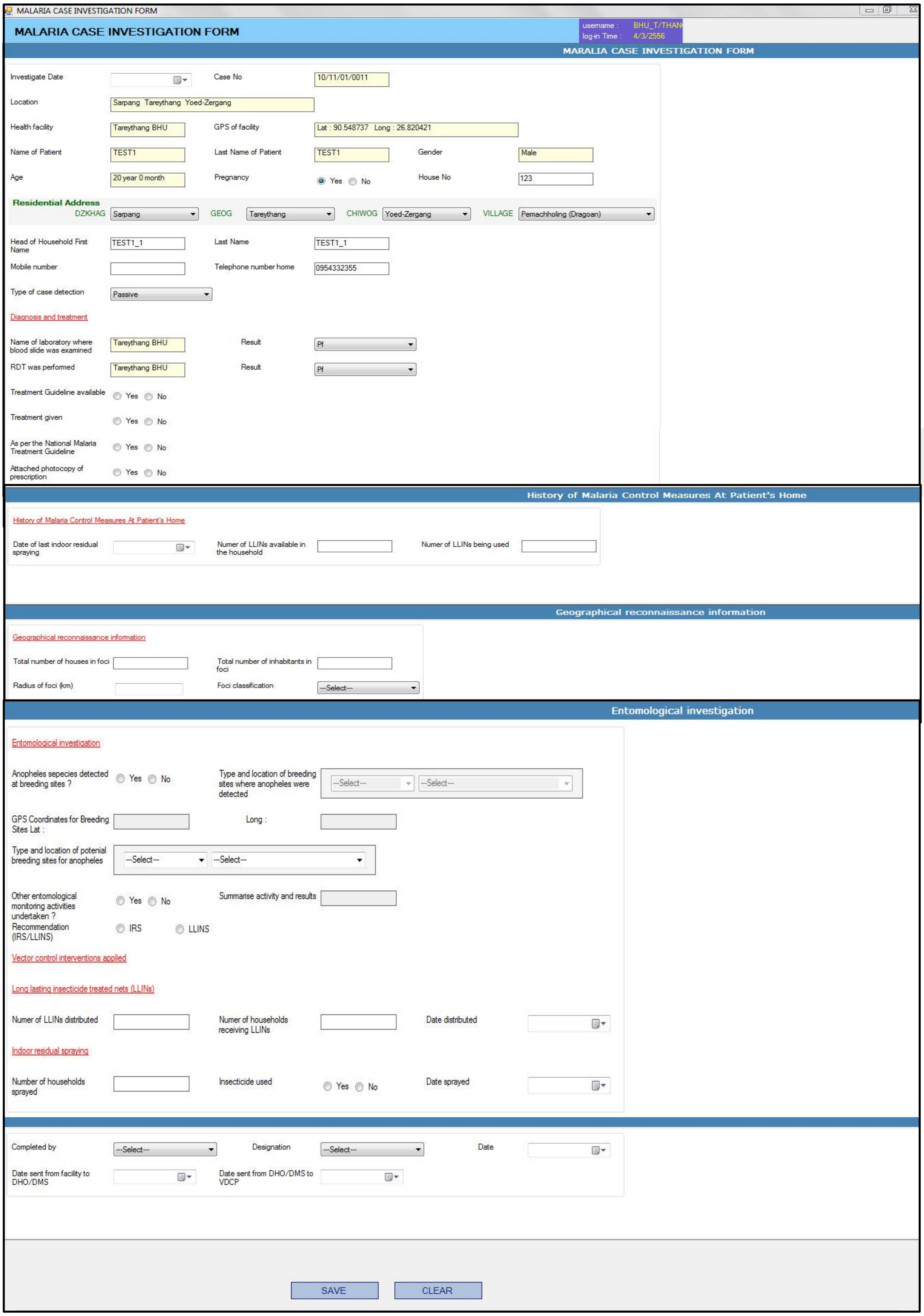
MALARIA CASE INVESTIGATION FORM

MALARIA CASE INVESTIGATION FORM

username : BHU_T/THAN
log-in Time : 4/3/2556

MARALIA CASE INVESTIGATION FORM

Investigate Date

Case No 10/11/01/0011

Location Sarpang Tareythang Yoed-Zergang

Health facility Tareythang BHU

GPS of facility Lat : 90.548737 Long : 26.820421

Name of Patient TEST1

Last Name of Patient TEST1

Gender Male

Age 20 year 0 month

Pregnancy Yes No

House No 123

Residential Address

DZKHAG Sarpang GEOG Tareythang CHIWOG Yoed-Zergang VILLAGE Pemachholing (Dragoan)

Head of Household First Name TEST1_1

Last Name TEST1_1

Mobile number

Telephone number home 0954332355

Type of case detection Passive

Diagnosis and treatment

Name of laboratory where blood slide was examined Tareythang BHU

Result Pf

RDT was performed Tareythang BHU

Result Pf

Treatment Guideline available Yes No

Treatment given Yes No

As per the National Malaria Treatment Guideline Yes No

Attached photocopy of prescription Yes No

History of Malaria Control Measures At Patient's Home

History of Malaria Control Measures At Patient's Home

Date of last indoor residual spraying

Numer of LLINs available in the household

Numer of LLINs being used

Geographical reconnaissance information

Geographical reconnaissance information

Total number of houses in foci

Total number of inhabitants in foci

Radius of foci (km)

Foci classification --Select--

Entomological investigation

Entomological investigation

Anopheles sepecies detected at breeding sites ? Yes No

Type and location of breeding sites where anopheles were detected --Select-- --Select--

GPS Coordinates for Breeding Sites Lat :

Long :

Type and location of potenial breeding sites for anopheles --Select-- --Select--

Other entomological monitoring activities undertaken ? Yes No

Summarise activity and results

Recommendation (IRS/LLINS) IRS LLINS

Vector control interventions applied

Long lasting insecticide treated nets (LLINs)

Numer of LLINs distributed

Numer of households receiving LLINs

Date distributed

Indoor residual spraying

Number of households sprayed

Insecticide used Yes No

Date sprayed

Completed by --Select--

Designation --Select--

Date

Date sent from facility to DHO/DMS

Date sent from DHO/DMS to VDCP

SAVE CLEAR

(Figure 13)

3.4 Case follow-up

This menu will be used to enter data of malaria case follow-up, and this menu will be linked with follow-up application on mobile phone. Generally, malaria case in Bhutan will be followed up 3 times at day 3, day, 14 and day 28. However, users can directly enter data on malaria case follow-up form by click on date in column of appointment date (Figure 14) and then the widow of malaria case follow-up form will be opened and users can enter follow-up's data by following each fields in the form include; clinical status, blood test for malaria parasites, medication administration *(day 3 and day 14 only)*, and lost to follow-up (Figure 15).

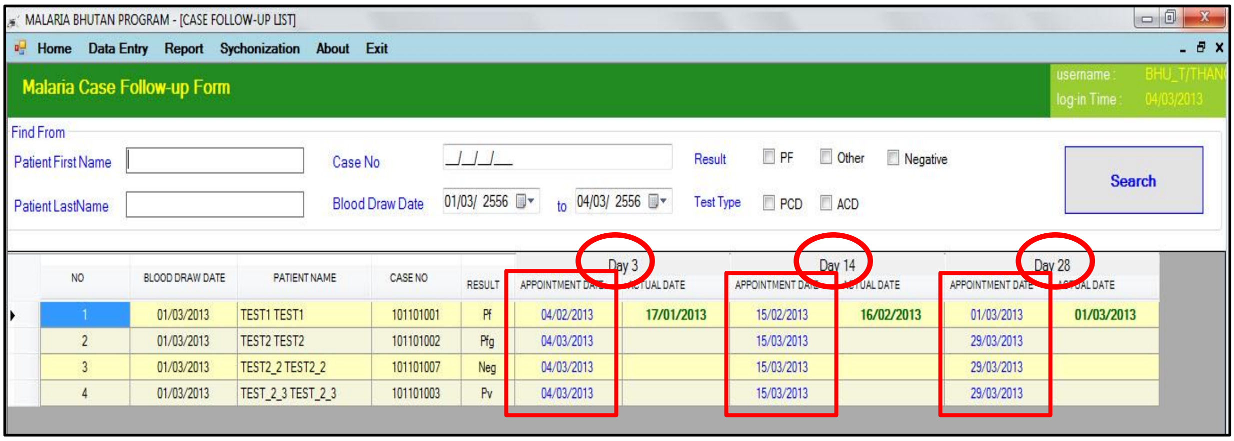

(Figure 14)

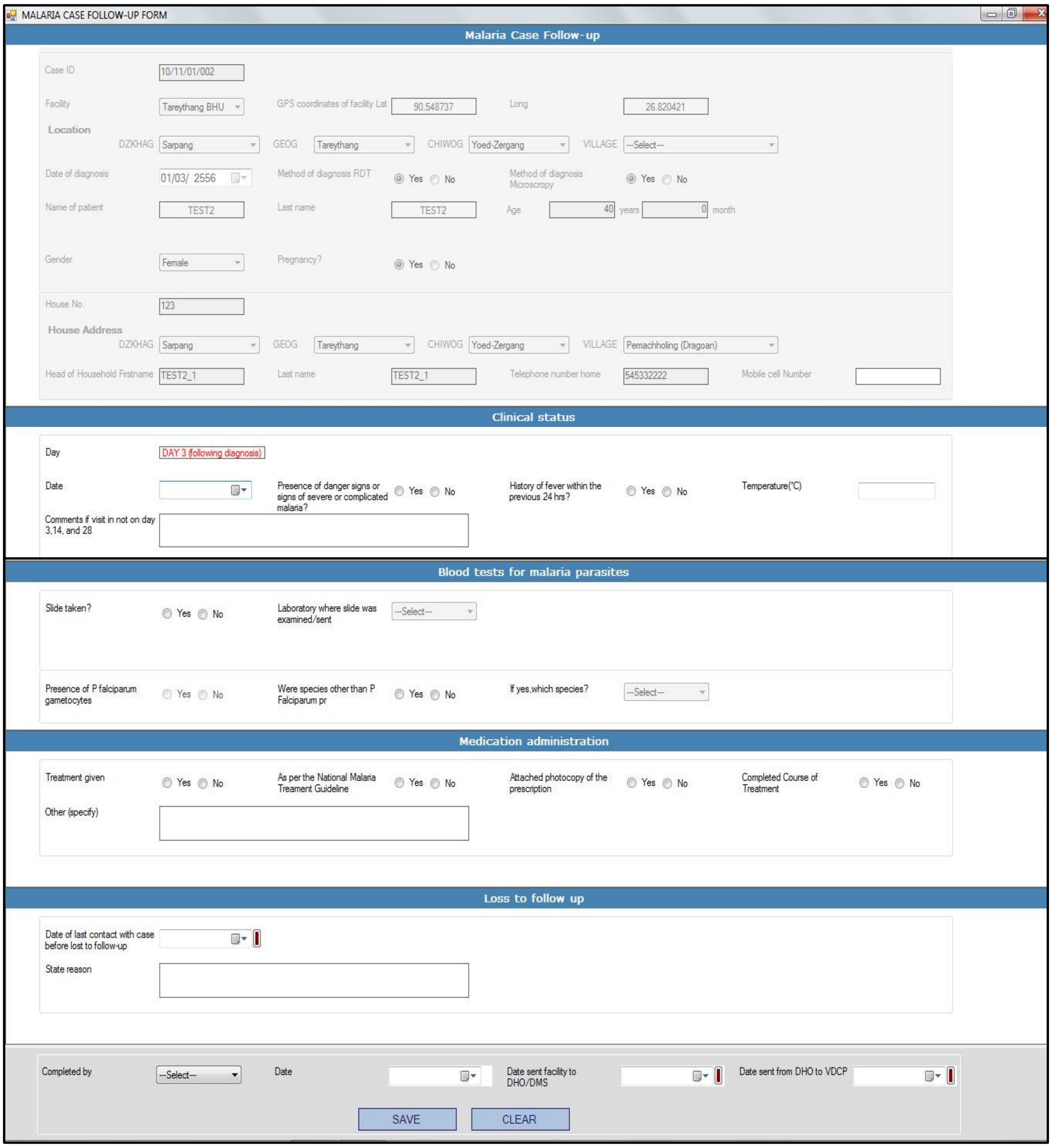

(Figure 15)

4. REPORT

This menu will be used to see and export report of all malaria case form. This menu composes of 5 report of malaria case form include;

- Malaria case registration form
- Malaria case notification form
- Malaria case investigate form
- Malaria case follow-up form
- Fever report form

4.1 Malaria case registration form

In this menu, users can see and export report of malaria laboratory register. To print report in standard format, users have to choose the range of time that users want to know and then click **"PRINT"**[1] to print out report of summary malaria laboratory register during time which users selected (Figure 16). Users also export report in excel format by click at **"EXPORT to EXCEL"** [2] and then choose destination to save this report.

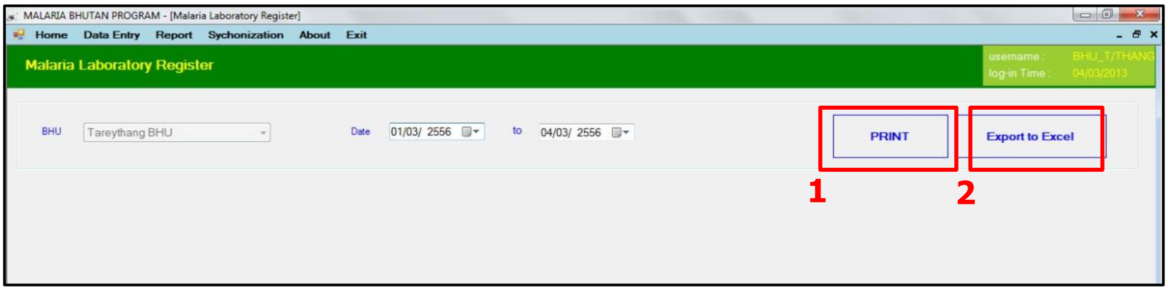

(Figure 16)

4.2 Malaria case notification form

This menu will be used to see and export individual report of malaria case notification form. Firstly, users have to selected conditions for searching malaria cases then list of malaria case will be showed in table. Users can see report one by one by click **"PREVIEW"** key at the end of malaria case's row in table and print out report. Users also export report in excel format by click at **"EXPORT to EXCEL"** and then choose destination to save this report (Figure 17).

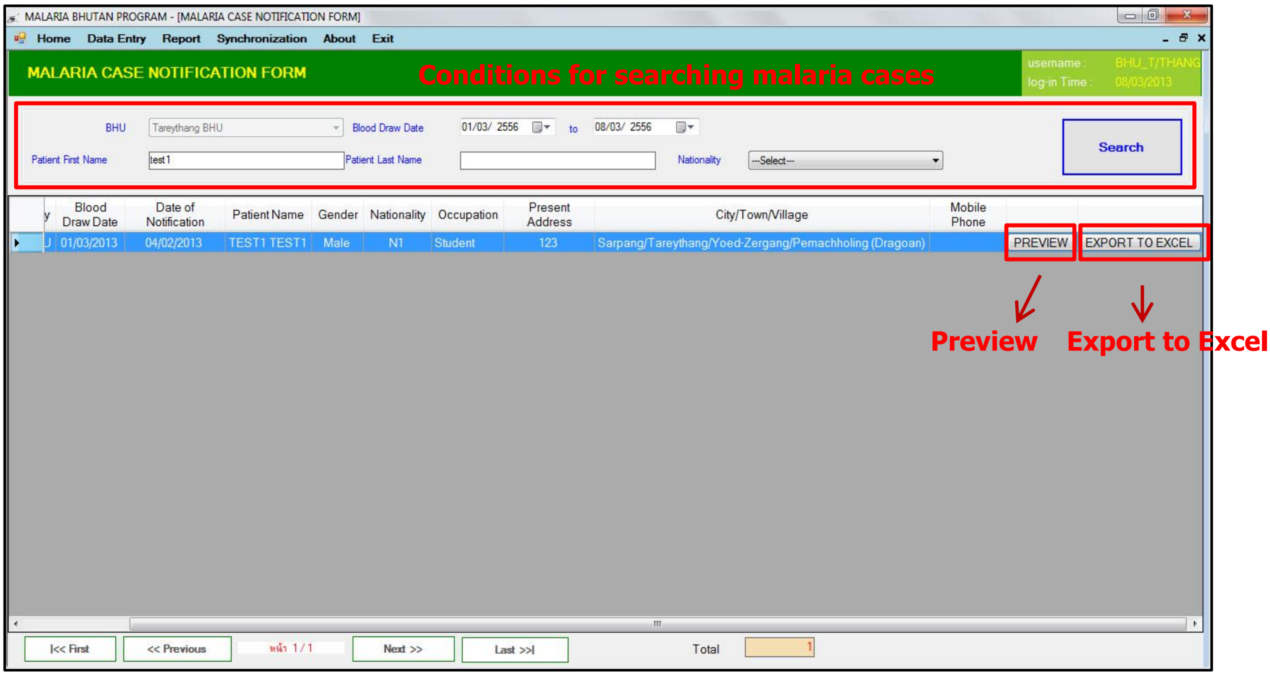

(Figure 17)

The report of malaria case notification form is showed below;

VDCP/SUR/F-01

MALARIA CASE NOTIFICATION FORM

MINISTRY OF HEALTH

VECTOR-BORNE DISEASE CONTROL PROGRAMME

DISTRICT Sarpang **Geog** Tareythang **Date** 04 / 02 / 2013

Name of Facility Tareythang BHU

GPS coordinates of facility **Case No.**

(2 District,2 geog,2 chiwog,3 patient No.)

Lat 26.82 Long 90.55

Please indicate if: In patient [X] or Out patient [ ]

Is the patient going to be referred? Yes [ ] No [X]

If yes,name of facility patient referred to Tareythang BHU

……..For Example……..

PATIENT INFERMATION

Patient Name TEST1 TEST1 Gender Male [X] Female [ ]

Age 20 years 0 months

Nationality

ID No: Mobile phone number

Permanent address: Town/Village

Head of Household/family

Telephone number home Mobile number (head of HH)

Present Address (if different than above 123

City/Town/Village Sarpang , Tareythang , Yoed-Zergang , Pemachholing (l Head of Household name TEST1_1 TEST1_1

Telephone number home 0954332355 Mobile number (head of HH)

Current Occupation Student Work Address

History of Blood transfusion Yes [X] No [ ] If Yes Date 23 / 01 / 2013

If working away from village of residence,how Daily [X] Weekly [ ] Monthly [ ] Annually [ ] Any other [ ]

Page 1 case notification form

HISTORY

Date of onset of illness/fever 30 / 01 / 2013

Where did the patient sleep during the period before the illness(tick where appropriate)

									Address,if different from above:
0-7 Days	Home	☐	Field	☐	Cattle shed	☐	Other	☐	
8-14 Days	Home	☐	Field	☐	Cattle shed	☐	Other	☐	
18-21 Days	Home	☐	Field	☐	Cattle shed	☐	Other	☐	
21-42 Days	Home	☐	Field	☐	Cattle shed	☐	Other	☐	
42 Days - 1 yr	Home	☐	Field	☐	Cattle shed	☐	Other	☐	

Has the patient travelled to a malaria endemic district/area within the last 14 day Yes [X] No [ ]

If yes, whrer did the patient travel?

Date(s) of travel From 01 / 12 / 2012 to 07 / 12 / 2012

Has the patient traveled to any country outside in the last 14 days? Yes [X] No [ ]

If yes,where (state name of country) Nepal

Date(s) of travel From 01 / 12 / 2012 to 19 / 12 / 2012

Type of preventative measures taken before and during travel MOSQUITO REPELLANTS

i.e. LLINs,mosquito repellants,etc)

Is the patient going to travel to another area within the next 42 days? Yes [X] No [ ]

If yes,provide exact places to be visited

DIAGNOSIS AND TREATMENT

Method of diagnosis **RDT** Date performed 01 / 03 / 2013

Name of officer conducting RDT

RDT result given Positive [X] Negative [ ]

Microscopy Date performed 01 / 03 / 2013 Date smear examined / /

Slide No: 1 Date result received / /

BS result given Positive [X] Negative [ ]

Gametocytes present in smear? Yes [ ] No [X]

Name of examining facility Tareythang BHU

Name & designation of the examining office

Page 2 case notification form

.........For Example.........

ACTION TO BE TAKEN (i.e. case investigation,contact screening,etc

Reported By BHU_T/THANG BHU_T/THANG Designation

Date 28 / 02 / 2013 Signature

Date sent from facility to DHO/DMS 11 / 02 / 2013

Date sent from DHO/DMS to VDCP 23 / 02 / 2013

FOR OFFICIAL USE BY VDC PROGRAMME ONLY Case No.

Date report received by VDC Programme / /

location of infection GPS coordinates of case Lat .00 Long .00

Name of examining facility

Name of examining officer

Case classification code number 0 Link to another case? Yes ☐ No ☐

....................For Example......................

Key for classification

Code	Classification
1	Imported case - contracted outside Bhutan
2	Imported case - contracted within Bhutan but in another locality
3	Imported case - contracted from an imported case (outside Bhutan)
4	Imported case - secondary case,contracted from an imported case (Local endemic areas)
5	Imported case - local case,contracted from an introduced or induced case
6	Imported case - contracted locally (Blood transfution)
7	Imported case - local (indigenous) case from within the last 6 months

Page 3 case notification form

4.3 Malaria case investigate form

This menu will be used to see and export individual report of malaria case investigation form. Firstly, users have to selected conditions for searching malaria cases then list of malaria case will be showed in table. Users can see report one by one by click **"PREVIEW"** key at the end of malaria case's row in table and print out report. Users also export report in excel format by click at **"EXPORT to EXCEL"** and then choose destination to save this report (Figure 18).

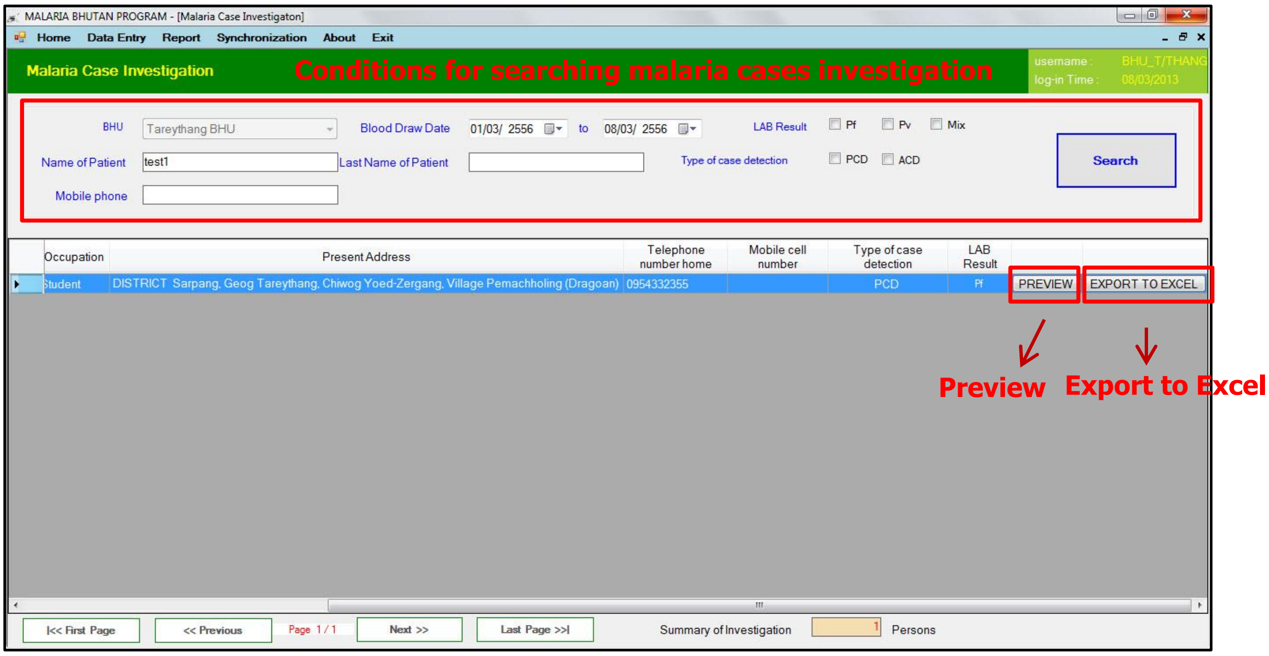

(Figure 18)

The report of malaria case investigation form is showed below;

MALARIA CASE INVESTIGATION FORM VDCP/SUR/F-02

MINISTRY OF HEALTH

VECTOR-BORNE DISEASE CONTROL PROGRAMME

DISTRICT Sarpang **Geog** Tareythang Date of investigation ....

Name of Facility Tareythang BHU GPS coordinates of facility Lat 26.82 Long 90.55

Case ID ....../....../....../......

(9 digits) District No geog No chiwog No Case No (3 digits)

Name of patient TEST1 TEST1

Age .... Gender Male [X] Female [ ]

Residential Address 123 Pregnant? Yes [X] No [ ]

Geog/Town/Village Sarpang/Tareythang/Yoed-Zergang/Pemachholing (Dragoan)

Head of Household name/family TEST1_1 TEST1_1 Mobile cell Number ....

Telephone number home 0954332355

Type of case detection (i.e. how case was first identifie

Passive [X] Active [ ] Other (survey,Research etc) [ ]

Specify ....

Diagnosis and treatment

Name of labratory where blood slide was examined Tareythang BHU Result Pf

RDT was performed .... Result Pf

Treatment Guideline available Yes [ ] No [ ]

Treatment given Yes [ ] No [ ]

As per the Nation Malaria Treatment Guideline Yes [ ] No [ ]

Attached photocopy of the prescription Yes [ ] No [ ]

Other (specify) ....

History of Malaria Control Measures At Patient's Hom **Page 1 Case investigation form**

Date of last indoor residual spraying ....

Number of LLINs available in the household .... Number of LLINs being used ....

Complete this section if the number of introduced cases exceeds two

.........For Example.........

Geographical reconnaissance informatio

Total number of houses in foci .... Total number of inhabitants in foc ....

Redius of foci .... Foci classification .... Check if m km

Entomological investigatio

Date of investigation ....

Anopheles species detected at breeding sites? Yes ☐ No ☐

Type and location of breeding sites where anopheles were detected ....

GPS Cordinates for Breeding Sites Lat .... Long ....

Type and location of potenial breeding sites for anopheles ....

....

Other entomological monitoring activities Yes ☐ No ☐

Summarise activity and results:

....

....

....

Recommendation (IRS/LLINS) ....

Vector control interventions applie

Long lasting insecticide treated nets (LLINs)

Number of LLINs Distributed .... Number of households receiving LLINs ....

Date distributed ....

Indoor residual spraying

Number of households sprayed .... Insecticide used ....

Date sprayed ....

Other intervention applied (explain)

....

Completed by .... Designation ....

Date .... Signature ....

Date sent from facility to DHO/DMS 11/02/2013 Date sent from DHO to VDCP 23/02/2013

.........For Example.........

Page 2 case investigation form

4.4 Malaria case follow-up form

This menu will be used to see and export individual report of malaria case follow-up form. Firstly, users have to selected conditions for searching malaria cases then list of malaria case will be showed in table. Users can see report one by one by click **"PREVIEW"** key at the end of malaria case's row in table and print out report. Users also export report in excel format by click at **"EXPORT to EXCEL"** and then choose destination to save this report (Figure 19).

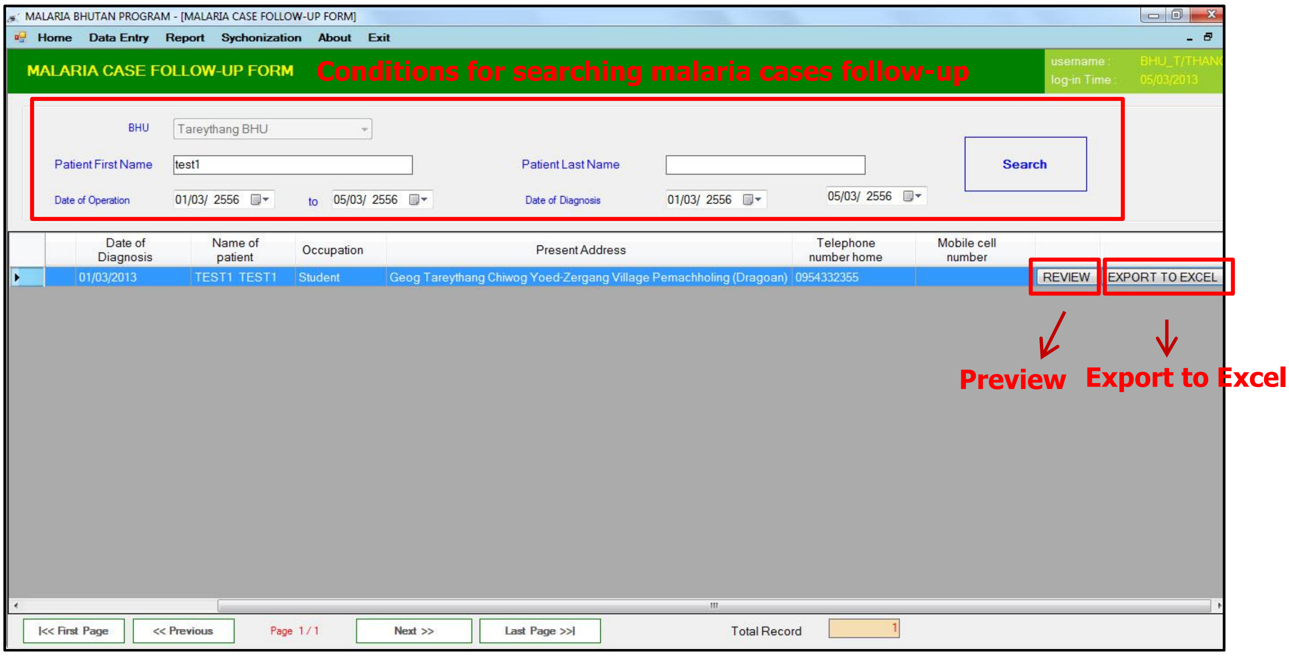

(Figure 19)

The report of malaria case follow-up form is showed below;

MALARIA CASE FOLLOW-UP FORM VDCP/SUR/F-03

MINISTRY OF HEALTH

VECTOR-BORNE DISEASE CONTROL PROGRAMME

DISTRICT Sarpang **Geog** Tareythang **Chiwog** Yoed-Zergang

Facility Tareythang BHU Date 01/03/2013

GPS coordinates of facilit Lat 26.82 Long 90.55 Case ID 101101001

(9 digits) District No geog No chiwog No Case No (3 digits)

Date of Diagnosis 01/03/2013

Method of diagnosis Microscopy X RDT X

Name of patient TEST1 TEST1 Age 563 Years 0 Months

Gender Male X Female

Present Address 123 Pregnant? Yes X No

Gewog/Village Tareythang/Yoed-Zergang/Pemachholing (Dragoan)

Head of Household name TEST1_1 TEST1_1

Telephone number home 0954332355 Mobile cell Number

Clinical statu

Day 3 (Following diognosis

Presence of danger signs or signs of severe or complicated malaria? Yes X No Date 17/01/2013

History of fever within the previous 24 hrs? Yes X No Temperature(axillary) 25.00 degrees celcius

Day 14 (Following diognosis)

Presence of danger signs or signs of severe or complicated malaria? Yes X No Date 16/02/2013

History of fever within the previous 24 hrs? Yes X No Temperature(axillary) 20.00 degrees celcius

Day 28 (Following diognosis)

Presence of danger signs or signs of severe or complicated malaria? Yes X No Date 01/03/2013

History of fever within the previous 24 hrs? Yes X No Temperature(axillary) 20.00 degrees celcius

Comment if visit is not on day 3,14 and 28

Blood tests for malaria parasite

Day 3 (Following diognosis

Slide taken? Yes X No Laboratory where slide was examined/sent Tareythang BHU

Blood Slide results

Asexual P falciparum parasitimea (/µl)

Presence of P falciparum gametocytes Yes X No Were species other than P Falciparum pr Yes X No

Page 1 case follow up form If yes,which species? P vivax X P ovale P malaria

.......For Example.........

Day 14 (Following diognosis

Slide taken? Yes [X] No [ ] Laboratory where slide was examined/sent Tareythang BHU

Slide results

Asexual P falciparum parasitimea (/µl) ....

Presence of P falciparum gametocytes Yes [X] No [ ] Were species other than P Falciparum present ? Yes [ ] No [X]

If yes,which species? P vivax [ ]

Day 28 (Following diognosis

Slide taken? Yes [X] No [ ] Laboratory where slide was examined/sent Tareythang BHU

Slide results

Average number of asexual P falciparum parasites (/µl ....

Presence of P falciparum gametocytes Yes [X] No [ ] Were species other than P Falciparum present ? Yes [ ] No [X]

If yes,which species? P vivax [ ]

Medication administration

	Yes		No	
Treatment given:	Yes	X	No	
As per the National Malaria Treatment Guideline	Yes	X	No	
Attached photocopy of the presciption	Yes	X	No	
Complete Course of Treatment	Yes	X	No	

Other (specify) ....

Loss to follow up

Date of last contact with case before lost to follow-up : ....

Reasons for loss to follow-u

State reasons ....

Completed by .... Designation ....

Date 01/03/2013 Signature ....

Date sent from facility to DHO/DMS .... Date sent from DHO to VDCP ....

....................For Example....................

Page 2 case follow up form

4.5 Fever report form

This menu will be used to see and export report of fever report form. Firstly, users have to selected week for searching malaria fever cases. Then, users can see report by click **"PRINT"** key to print out report. Users also export report in excel format by click at **"EXPORT to EXCEL"** and then choose destination to save this report (Figure 20).

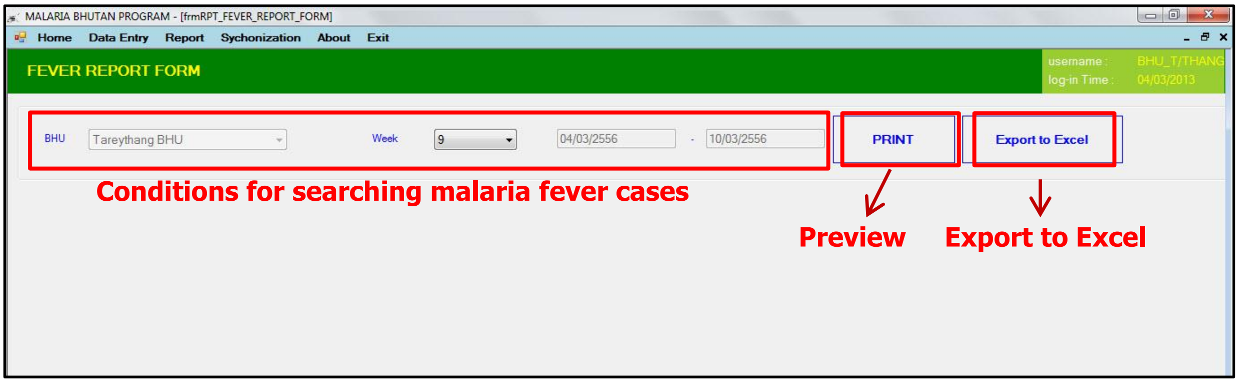

(Figure 20)

The malaria fever case report form is showed below;

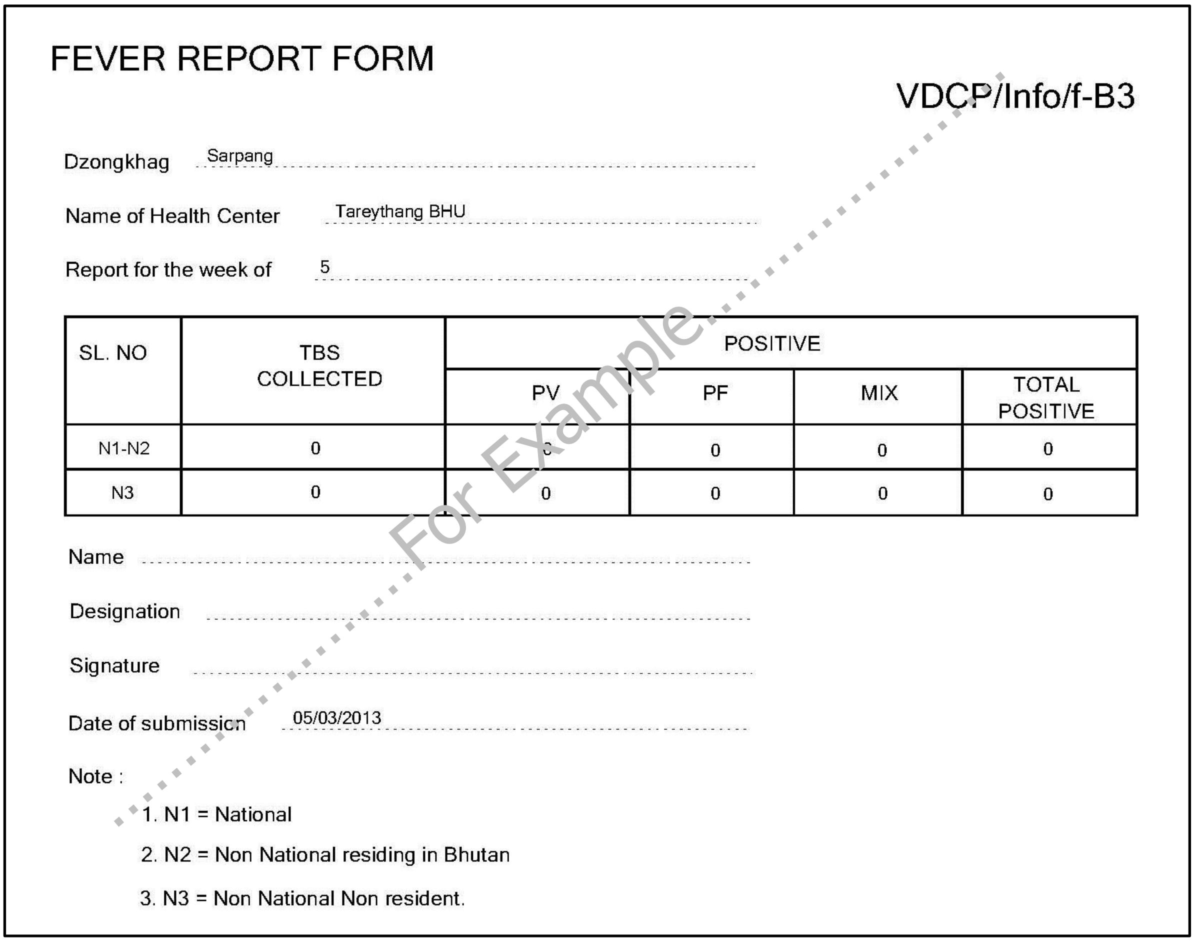

FEVER REPORT FORM

VDCP/Info/f-B3

Dzongkhag Sarpang

Name of Health Center Tareythang BHU

Report for the week of 5

SL. NO	TBS COLLECTED	POSITIVE			
		PV	PF	MIX	TOTAL POSITIVE
N1-N2	0	0	0	0	0
N3	0	0	0	0	0

Name

Designation

Signature

Date of submission 05/03/2013

Note :

1. N1 = National
2. N2 = Non National residing in Bhutan
3. N3 = Non National Non resident.

For Example

5. SYNCHRONIZATION

After users entered data for each form into the program, this menu will be used to synchronize all data to the data center. To synchronize all data to the data center, users can click **"SYNCHRONIZATION"** menu at the menu bar (Figure 21) then click **"SYNCHRONIZE DATA"** and all data will be synchronized to the data center. This process will take several minutes. When all data was already synchronized, the system will show warning message.

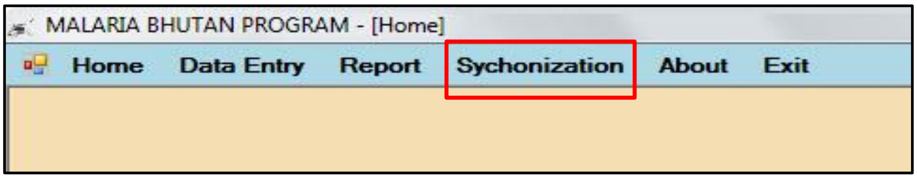

(Figure 21)

6. ABOUT

The menu will show detail of the **"Bhutan Malaria System"** (Figure 22).

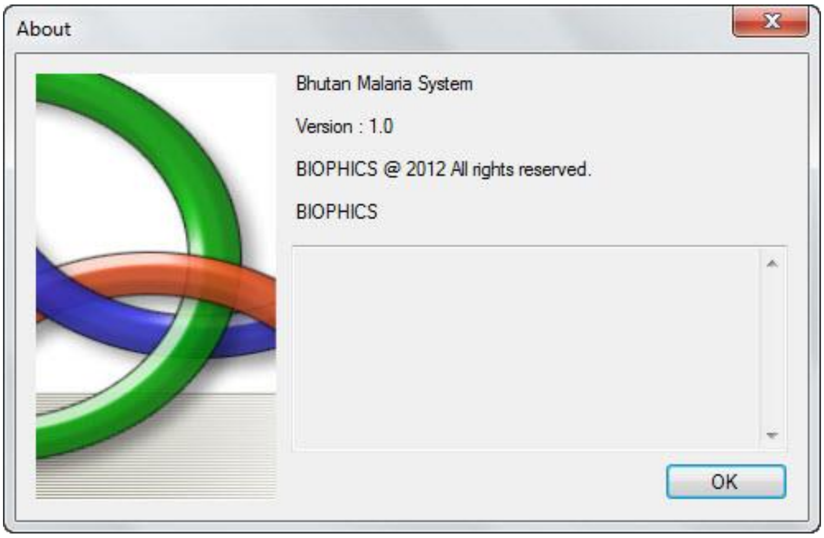

(Figure 22)
