# Supplementary material for: Performance and user acceptance of the Bhutan febrile and malaria information system: report from a pilot study
Source: Malar J. 2016 Jan 29;15:52. doi: 10.1186/s12936-016-1105-0 (PMC4731940; doi:10.1186/s12936-016-1105-0)
Supplement: Supplementary file 2 — 10.1186/s12936-016-1105-0 Quantitative questionnaire assessing acceptance and ease of use of the BFMIS (Form A). [file 12936_2016_1105_MOESM2_ESM.docx]

**Additional Material 2:** Quantitative questionnaire assessing acceptance and ease of use of the BFMIS (Form A)

| **Section A. Demographic** |
| --- |

1. Sex:………..
2. Age:…….…..
3. Position:……………………………...
4. Designation:……………………………………………
5. Office/Unit:……………………………………
6. Health Facility:.....................................................
7. How long have you been using the system? …….……year …………months

| **Section B : User Acceptance of the system function** |
| --- |

Please think your experiences in using the system and give a rating *as follow:*

*1= Strongly Disagree,*

*2 =Agree,*

*3= Neither Disagree nor Disagree,*

*4 = Agree,*

*5= Strongly Agree*

| ***Perceived ease of use*** | **1** | **2** | **3** | **4** | **5** |
| --- | --- | --- | --- | --- | --- |
| The user interface is friendly and easy to use |  |  |  |  |  |
| System navigation is intuitive and easy to use |  |  |  |  |  |
| It is easy to login and generates the report |  |  |  |  |  |
| Learning to operate the program was easy |  |  |  |  |  |
| Procedure were simple and required a minimum number of steps |  |  |  |  |  |
| Errors were easy to recover from. The error message gave useful information |  |  |  |  |  |
| The interface, menu and screen, were laid out in a logical fashion |  |  |  |  |  |
| The prompts and error messages were appropriate in tone (i.e. not degrading or condescending) |  |  |  |  |  |
| Command names and options made sense |  |  |  |  |  |
| Pacing was comfortable, neither too fast nor too slow |  |  |  |  |  |
| Command names and what the commands did were easy to remember |  |  |  |  |  |
| It was easy to find the commands and information required to complete the task |  |  |  |  |  |
| ***Perceived usefulness*** | **1** | **2** | **3** | **4** | **5** |
| Provided on-line help, tips and screen casts are useful |  |  |  |  |  |
| The system response is adequate to my requests |  |  |  |  |  |
| The automatically produce summary report give me a better overview of the textual content |  |  |  |  |  |
| It took a reasonable amount of time to complete most tasks |  |  |  |  |  |
| The computer did not take over the work or “get in the way” |  |  |  |  |  |
| Using this new system makes me do the job more efficient |  |  |  |  |  |
| There were few surprises. The computer worked as expected |  |  |  |  |  |
| Online help was informative so I can complete the work without worry |  |  |  |  |  |
| The user manual was informative such that I can work well |  |  |  |  |  |

| ***Overall Attitude*** | **1** | **2** | **3** | **4** | **5** |
| --- | --- | --- | --- | --- | --- |
| The system is responsive (overall impression) |  |  |  |  |  |
| The system increase your work experience |  |  |  |  |  |
| The system offers complete set of work functionalities |  |  |  |  |  |
| The system produces comprehensive multi information and output |  |  |  |  |  |

| ***Satisfaction*** | **1** | **2** | **3** | **4** | **5** |
| --- | --- | --- | --- | --- | --- |
| I am happy with the user interface of the system |  |  |  |  |  |
| I am satisfied with the online help |  |  |  |  |  |
| I am satisfied with the data entry function |  |  |  |  |  |
| I am satisfied with the data edit/management of the system |  |  |  |  |  |
| I am satisfied with the report generation |  |  |  |  |  |
| I am satisfied with the alert threshold |  |  |  |  |  |

**How significant are the following factors influencing your acceptance when implementing a new web-based system methodology or substantial change in the clinic/office?**

| **Statements** | **Very significant** | **Significant** | **Un-significant** | **Very Un-significant** |
| --- | --- | --- | --- | --- |
| Knowledge of the change before implementation |  |  |  |  |
| Previous experience of the implemented methodology |  |  |  |  |
| Available training |  |  |  |  |
| Consultation with you before change is implemented |  |  |  |  |
| Opinions of others |  |  |  |  |
| Output of work |  |  |  |  |

| **Section C : Problems and program errors occurring during the system use** |
| --- |

**Did you encounter any problems in using this web-based system? If yes, please check what kind of problems/errors you found?**

|  | **Yes** | **No** |
| --- | --- | --- |
| Program bug |  |  |
| Wrong calculation |  |  |
| Reporting output errors |  |  |
| Process time |  |  |
| **Other.** *Please specify:* | | |

| **Section D : Opinions about the system in term of continuing use and program improvement** |
| --- |

**Do you think that new system is better than the original paper-based system?**

🖵 Yes 🖵 No

**If you can choose, will you use the new system or not?**

🖵 Yes 🖵 No

**If NOT - why?**

🖵 The benefit of the web-based system would not be apparent.

🖵 The web based system make my job more difficult.

🖵 Other specify:……………………………………………...

………………………………………………

**From your experiences using the new system, which functions that you would like to get the system improve?**

| **Function** | **Yes** | **No** |
| --- | --- | --- |
| Menu |  |  |
| Data entry function |  |  |
| Data management |  |  |
| Data quality check |  |  |
| Reporting |  |  |
| Other. *Please specify:* | | |

**Overall comments suggestions about this system**

………………………………………………………………………………………………………

………………………………………………………………………………………………………

………………………………………………………………………………………………………

………………………………………………………………………………………………………

………………………………………………………………………………………………………

**Thank you for your kind cooperation!**
