# Supplementary material for: Performance and user acceptance of the Bhutan febrile and malaria information system: report from a pilot study
Source: Malar J. 2016 Jan 29;15:52. doi: 10.1186/s12936-016-1105-0 (PMC4731940; doi:10.1186/s12936-016-1105-0)
Supplement: Supplementary file 3 — 10.1186/s12936-016-1105-0 Quantitative questionnaire assessing future usage (Form B). [file 12936_2016_1105_MOESM3_ESM.docx]

**Additional Material 3:** Quantitative questionnaire assessing future usage (Form B)

| **Section A. Demographic** |
| --- |

**About your clinic/office**

1. Name of the health facility:___________________________
2. Number of person currently working in the clinic/office ___________________
3. Computer system currently used in your clinic/office

□ None (Go to Question number 8)

□ Yes (Answer all that apply)

□ Desktop /Laptop, number ___________________

□ Server, number ___________________

□ Other (Please specify): _________________, number________________

1. Type of software being used in your office. (Answer all that apply)

□ Word processor (e.g. Microsoft Word)

□ Spreadsheet (e.g. MS Excel)

□ Presentation (e.g. MS Powerpoint)

□ Database software (e.g. MS Access)

□ Internet Browser (e.g. Internet Explorer, Safari)

□ E-mail Software (e.g. Outlook)

□ Other (Please Specify) ________________

1. Any Internet connection(s) in your office/clinic?

□ Don’t know (Go to Question number 6)

□ No (Go to Question number 6)

□ Yes via (Answer all that apply)

□ Dial up Modem

□ ADSL

□ Mobile broadband connection via a portable device using mobile telephone

networks (so called 3G or 4G)

□ Other (Please Specify) ________________

1. Who is your office’s Internet Service Providers? (Answer all that apply)

□ Don’t know

□ Paid ISP, Name ________________

1. How do you get access to the Internet? (Answer all that apply)

□ Never access the Internet

□ At the clinic

□ At the office

□ At home

□ Other (Please Specify) ________________

1. Number of person in your clinic/office routinely using computers: ___________

**About yourself**

1. Gender (select one):

□ Female □ Male

1. Age: ___________________
2. What is your highest level of education? (Check the appropriate response.)

□ Ph.D.

□ Master

□ Bachelor

□ Diploma

□ High School

□ Other (Please specify.) ___________________

1. How long have you been working in the health care field? (Please select one)

□ Less than 1 year

□ 1 – 3 years

□ 4 - 6 years

□ More than 6 years

1. How long have you been working in this clinic/office? (Please select one)

□ Less than 1 year

□ 1 – 3 years

□ 4 - 6 years

□ More than 6 years

13. What is your designation in health care field?

□ Doctor

□ Nurse

□ Midwife

□ Lab Technician

□ Other (Please specify) _________________

1. What is your average percent of time spent in the following activities?

□ Providing health care _________

□ Collecting and entering data _________

□ Preparing mandatory routine reports _________

□ Management and administration _________

□ Other (Please specify.) _________

1. If Electronic Medical/Health Record install, do you need any training?

□ Yes

□ No

| **Section B. Computer Usage** |
| --- |

1. How long ago did you first start using a computer?

□ Never Use

□ < 1 year

□ 1-2 years

□ > 2 years

1. How often do you use the computer?

□ Don’t use at all

□ Use <= 1 time each week

□ Use several times each week

□ Use about once a day

□ Use several times each day

1. To what extent do you personally use a computer for each of the following professional tasks? Please circle the answer.

| **Statements** | 1.  **Never perform this task** | 2. **Perform** this task but **never** use a computer | 3.  **Some-times** use the computer | 4.  **Often** use a computer | 5. **Always** use a computer |
| --- | --- | --- | --- | --- | --- |
| a. Recording patient data (e.g. clinical diagnosis, history, laboratory) |  |  |  |  |  |
| b. Retrieving patient clinical record (e.g. treatment, family planning) |  |  |  |  |  |
| c. Medical Treatment |  |  |  |  |  |
| d. Performing statistical analysis |  |  |  |  |  |
| e. Generating mandatory reports |  |  |  |  |  |
| f. Preparing presentation slides |  |  |  |  |  |
| g. Communicating with colleagues (e.g. E-mail) |  |  |  |  |  |
| h. Searching for information associated with office tasks (e.g. health literatures, government documents, etc.) |  |  |  |  |  |
| i. Searching for information associated with personal interest (e.g. news) |  |  |  |  |  |

1. What kind of training related to computer skilled have you had and how long?(Answer all that apply)

□ Formal school (University) computer

and related field courses training ___________________

□ Formal workshop, short courses ___________________

□ Self-guided learning about computers ___________________

□ Other way of training (Please specify.) ___________________

□ None

20. Have you ever used mobile phone or tablets to collect data in your work?

□ No

□ Yes

If yes, what program you use to collect data on mobile phone or tablet?

(please specify) ________________________________________

**Section C. Information and Communication Technology Knowledge**

Below are questions that relate to basic ICT knowledge. Please select only one answer for each question.

| Statements | True | False | Do Not Know |
| --- | --- | --- | --- |
| 1. Window is an example of an operating system. |  |  |  |
| 2. Software is a series of instruction that can make computer to do something. |  |  |  |
| 3. Computer virus is software that can damage computer. |  |  |  |
| 4. Scanner is an output device. |  |  |  |
| 5. LAN stands for Local Area Network. |  |  |  |
| 6. A computer network is the term used to describe a communication system connecting two or more computers together. |  |  |  |
| 7. The Internet is the network of worldwide computer networks. |  |  |  |
| 8. A data field is a component of a record |  |  |  |

| **Section D. User Acceptance** |
| --- |

**Computerized Health Record** in the survey stands for a computerized system of entering, managing and accessing the history of a patient’s health care within a single practice.

This part of the questionnaire asks for your opinion about CHR if there is a system available for you to use. We want to know how you expected or perceived ease of use, perceived usefulness and intention to use CHR system. Please indicate your degree of agreement by choosing only one response for each statement below.

| Statements | Strongly Agree | Agree | Neither Agree nor Disagree | Disagree | Strongly Disagree |
| --- | --- | --- | --- | --- | --- |
| **Perceived Ease of Use** | | | | | |
| a. My interaction with the CHR will be clear and understandable “user- friendly |  |  |  |  |  |
| b. Learning to use the CHR will be easy for me. |  |  |  |  |  |
| c. I expect to become skilled at using the CHR. |  |  |  |  |  |
| d. Overall, I expect the CHR will be easy for staff to use. |  |  |  |  |  |
| **Perceived Usefulness** | | | | | |
| a. Using the CHR will improve the quality of my work in providing better patient care. |  |  |  |  |  |
| b. Using the CHR will give me greater control over my work. |  |  |  |  |  |
| c. Using the CHR will allow me to accomplish tasks more quickly. |  |  |  |  |  |
| d. Using the CHR will allow me to accomplish more work than would otherwise be possible |  |  |  |  |  |
| e. Using the CHR will enhance my overall effectiveness in my job. |  |  |  |  |  |
| f. Using the CHR will make my job easier to perform. |  |  |  |  |  |
| g. Overall, the CHR should be a useful tool for practicing my profession. |  |  |  |  |  |
| **Intention to Use** | | | | | |
| a. If there is a CHR system available, I will definitely use CHR in my work |  |  |  |  |  |
| b. If there is a CHR system available, I intend to use CHR everyday |  |  |  |  |  |

.

**Thank you for your kind cooperation!**
