# Supplementary figures and images for: Performance and user acceptance of the Bhutan febrile and malaria information system: report from a pilot study
Source: Malar J. 2016 Jan 29;15:52. doi: 10.1186/s12936-016-1105-0 (PMC4731940; doi:10.1186/s12936-016-1105-0)

**Additional Material 4:** Perceived ease of use of the BFMIS

**
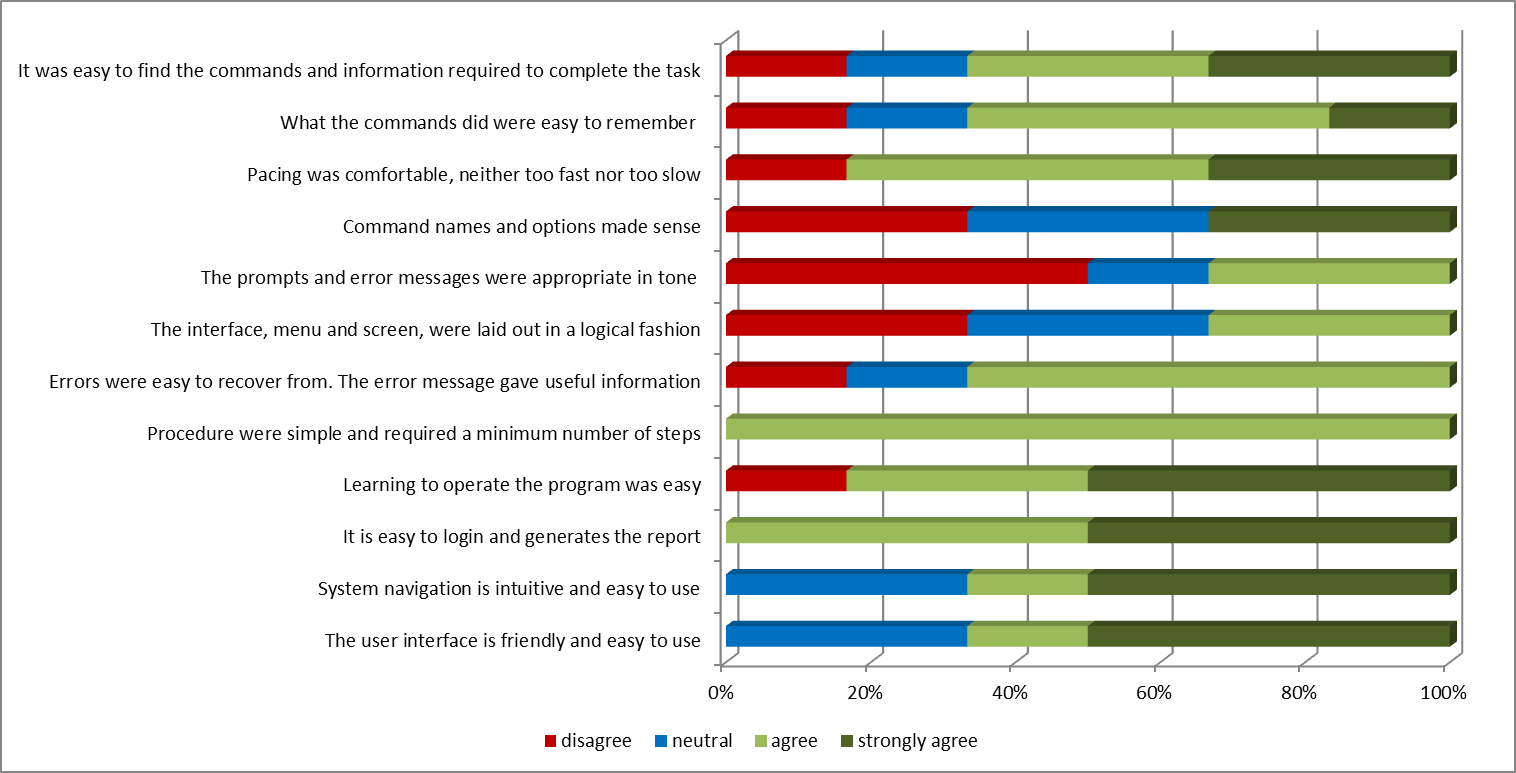
**

Supplement: Supplementary file 4 — 10.1186/s12936-016-1105-0 Perceived ease of use of the BFMIS. [file 12936_2016_1105_MOESM4_ESM.docx]

**Additional Material 5**: Perceived usefulness of the BFMIS

**
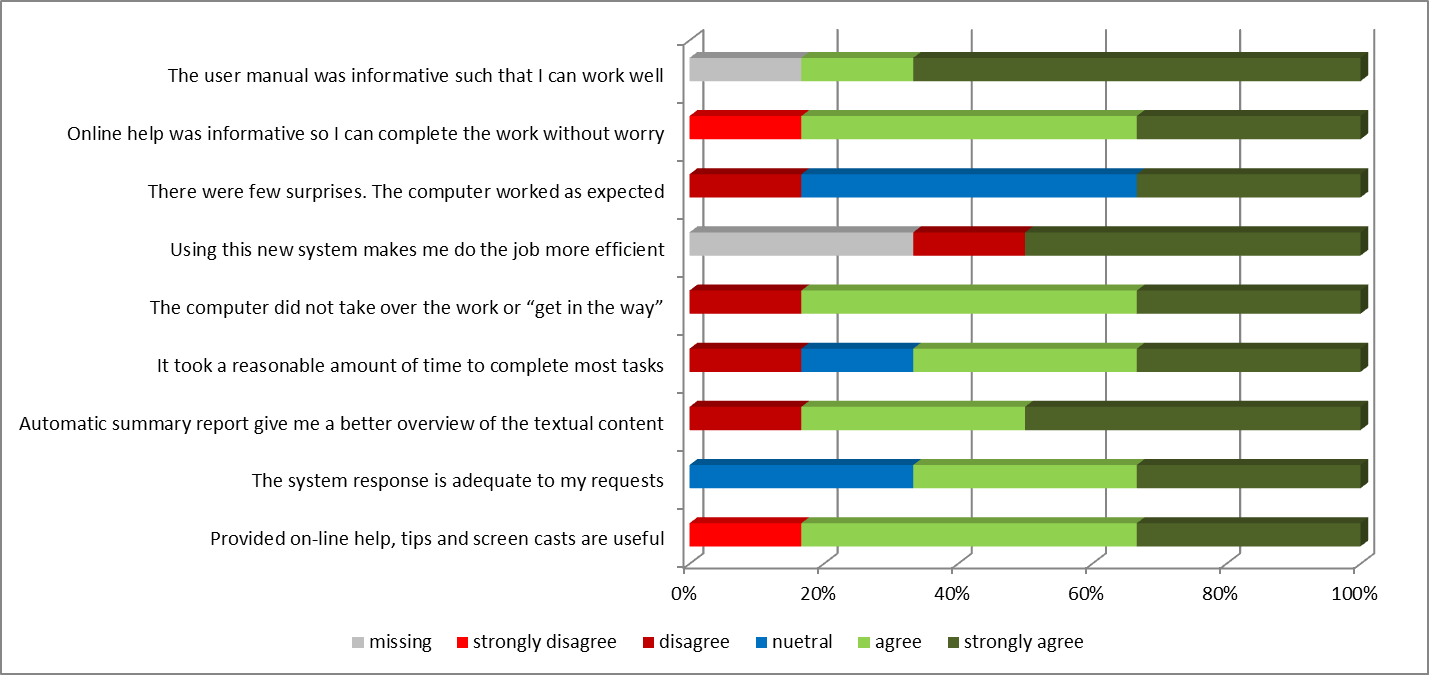
**

Supplement: Supplementary file 5 — 10.1186/s12936-016-1105-0 Perceived usefulness of the BFMIS. [file 12936_2016_1105_MOESM5_ESM.docx]

**Additional Material 7:** Overall Attitude, Satisfaction and Acceptance Factors of System Users


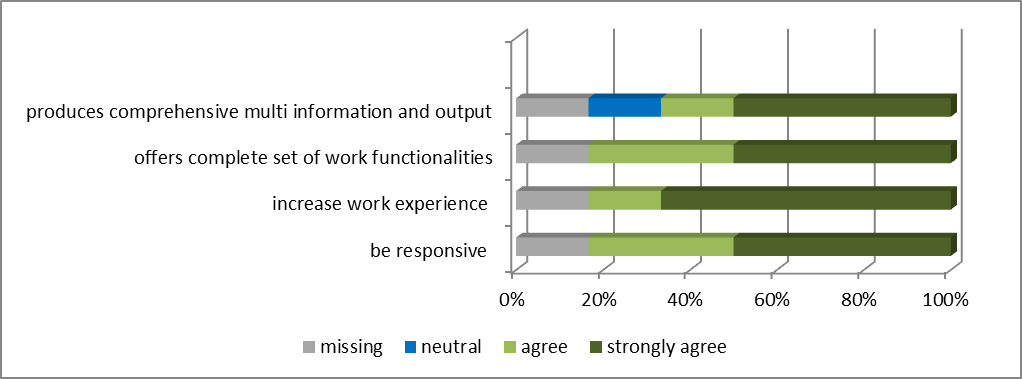


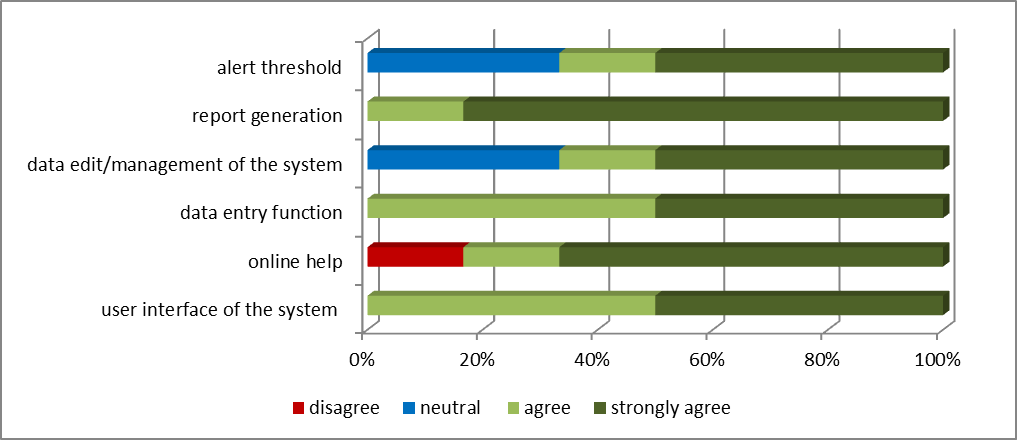


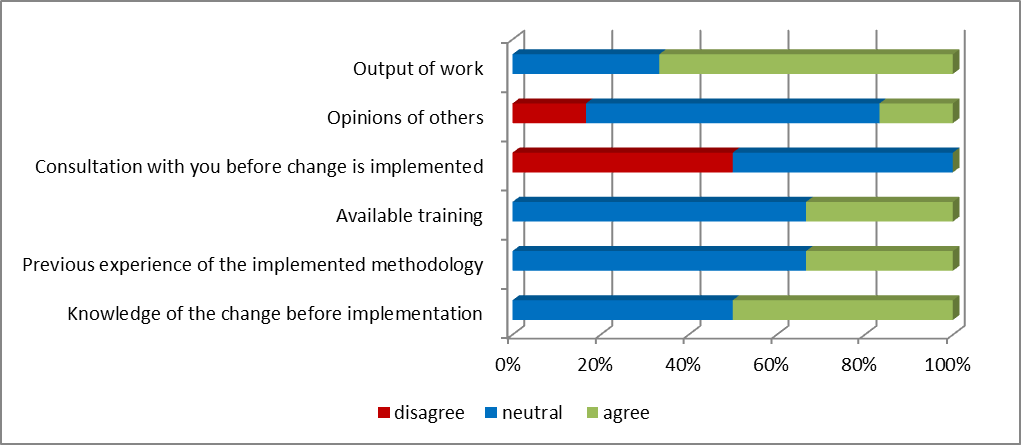

Supplement: Supplementary file 7 — 10.1186/s12936-016-1105-0 Overall Attitude, Satisfaction and Acceptance Factors of System Users. [file 12936_2016_1105_MOESM7_ESM.docx]
