# Supplementary material for: Performance and user acceptance of the Bhutan febrile and malaria information system: report from a pilot study
Source: Malar J. 2016 Jan 29;15:52. doi: 10.1186/s12936-016-1105-0 (PMC4731940; doi:10.1186/s12936-016-1105-0)
Supplement: Supplementary file 6 — 10.1186/s12936-016-1105-0 Feedback of BFMIS users at study sites. [file 12936_2016_1105_MOESM6_ESM.docx]

**Additional Material 6:** Feedbacks of BFMIS users at study sites

| **Feedbacks for the BFMIS** | n/N (%) |
| --- | --- |
| Problem(s) ever encountered using the system |  |
| Program bug | 1/6 (17%) |
| Wrong calculation | 1/6 (17%) |
| Reporting output errors | 3/6 (50%) |
| Process time | 2/6 (33%) |
| Functions of the system that should be improved |  |
| Menu | 3/6 (50%) |
| Data entry function | 3/6 (50%) |
| Data management | 3/6 (50%) |
| Data quality check | 5/6 (83%) |
| Reporting | 3/6 (50%) |

n,N = number of sites
